# Autophagy in the eye: from physiology to pathophysiology

**DOI:** 10.1080/27694127.2023.2178996

**Published:** 2023-03-01

**Authors:** Paloma B. Liton, Kathleen Boesze-Battaglia, Michael E. Boulton, Patricia Boya, Thomas A. Ferguson, Ian G. Ganley, Anu Kauppinnen, Gordon W. Laurie, Noboru Mizushima, Hideaki Morishita, Rossella Russo, Jaya Sadda, Rajalekshmy Shyam, Debasish Sinha, Debra A. Thompson, David N. Zacks

**Affiliations:** a Departments of Ophthalmology & Pathology, Duke School of Medicine, Duke University, Durham, NC 27705, USA; b Department of Basic and Translational Sciences, University of Pennsylvania, School of Dental Medicine, Philadelphia, PA 19104, USA; c Department of Ophthalmology and Visual Sciences, University of Alabama at Birmingham (UAB), Birmingham, AL, USA; d Department of Neuroscience and Movement Science. Faculty of Science and Medicine, University of Fribourg, 1700 Fribourg, Switzerland; e Department of Ophthalmology and Visual Sciences, Washington University in St. Louis, St. Louis, MO 63110, USA; f MRC Protein Phosphorylation and Ubiquitylation Unit, School of Life Sciences, University of Dundee, Dundee, DD1 5EH, UK; g Faculty of Health and Sciences, School of Pharmacy, University of Eastern Finland, 70210 Kuopio, Finland; h Departments of Cell Biology, Ophthalmology and Biomedical Engineering, University of Virginia, Charlottesville, VA 22908, USA; i Department of Biochemistry and Molecular Biology, Graduate School of Medicine, The University of Tokyo, 113-0033, Japan; j Department of Physiology, Juntendo University Graduate School of Medicine, Bunkyo-ku, Tokyo, 113-8421, Japan; k Preclinical and Translational Pharmacology, Glaucoma Unit, Department of Pharmacy, Health and Nutritional Sciences, University of Calabria, 87036 Rende, Italy; l Department of Ophthalmology and Visual Sciences, Kellogg Eye Center, University of Michigan Medical School, Ann Arbor, MI, USA; m School of Optometry, Indiana University, Bloomington, IN 47405, USA; n Department of Ophthalmology, Cell Biology, and Developmental Biology, University of Pittsburgh School of Medicine, Pittsburgh, PA, USA; o Wilmer Eye Institute, The Johns Hopkins University School of Medicine, Baltimore, MD, USA

**Keywords:** autophagy, eye, outflow pathway, trabecular meshwork, cornea, lens, photoreceptors, RPE, retinal epithelial cells, RGC, retina, retinal ganglion cells, glaucoma, eye diseases, AMD, corneal dystrophy, cataracts, vision cycle, lysosomes, dry eye, retinitis pigmentosa, retinal detachment

## Abstract

Autophagy is a catabolic self-degradative pathway that promotes the degradation and recycling of intracellular material through the lysosomal compartment. Although first believed to function in conditions of nutritional stress, autophagy is emerging as a critical cellular pathway, involved in a variety of physiological and pathophysiological processes. Autophagy dysregulation is associated with an increasing number of diseases, including ocular diseases. On one hand, mutations in autophagy-related genes have been linked to cataracts, glaucoma, and corneal dystrophy; on the other hand, alterations in autophagy and lysosomal pathways are a common finding in essentially all diseases of the eye. Moreover, LC3-associated phagocytosis, a form of non-canonical autophagy, is critical in promoting visual cycle function. This review collects the latest understanding of autophagy in the context of the eye. We will review and discuss the respective roles of autophagy in the physiology and/or pathophysiology of each of the ocular tissues, its diurnal/circadian variation, as well as its involvement in diseases of the eye.

## 1. Introduction

**Figure 1.**
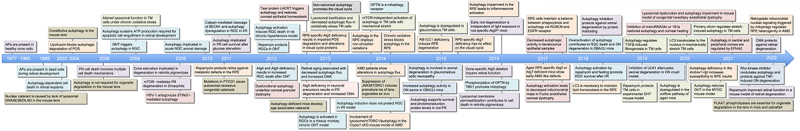
**Timeline of the most relevant milestones in autophagy research in the eye**. Each of the ocular tissues are represented by different color boxes. AP: autophagosomes; POS: photoreceptor outer segment; TM: trabecular meshwork; ONT: optic nerve transection; RGC: retinal ganglion cell; BECN1: beclin 1; I/R: ischemia/reperfusion; RPE: retinal pigment epithelium; PR: photoreceptor; HSV-1: Herpes Simple Virus-1; OPTN: optineurin; LACRT: lacritin; RB1CC1: RB1 Inducible Coiled-Coil 1; TBK1: TANK-binding kinase 1; CMA: chaperon-mediated autophagy; OHT: ocular hypertension; RUBCN: RUN domain and cysteine-rich domain containing Beclin 1-interacting protein; ON: optic nerve; AMD: age-related macular degeneration; ULK1: Unc-51 like autophagy activating kinase 1; MYOC: myocilin; EPHA2: EPH receptor A2; PLAAT: phospholipase A and acyltransferase.

Autophagy is a catabolic self-degradative pathway that involves the degradation of intracellular material through the lysosomal compartment. Autophagy was initially discovered under the context of starvation, and for many years it was believed its sole function was that of providing nutrients through the recycling of cell’s constituents. However, studies conducted during the last decade have shown that the role of autophagy goes beyond providing energy. Autophagy is emerging as a critical cellular pathway, which is involved in a variety of physiological processes to support cellular, tissue and organismal homeostasis. Consequently, autophagy dysregulation is associated to an increasing number of diseases, including diseases of the eye. A timeline of the early events and historical perspective of the major discoveries in autophagy research in the eye were previously collected in [1] and now updated in Figure 1. Here, we will review and discuss the respective roles of autophagy in the physiology and/or pathophysiology of each of the ocular tissues, and its implication in disease.

## 2. The Autophagy Pathway: Types and Mechanisms

**Figure 2.**
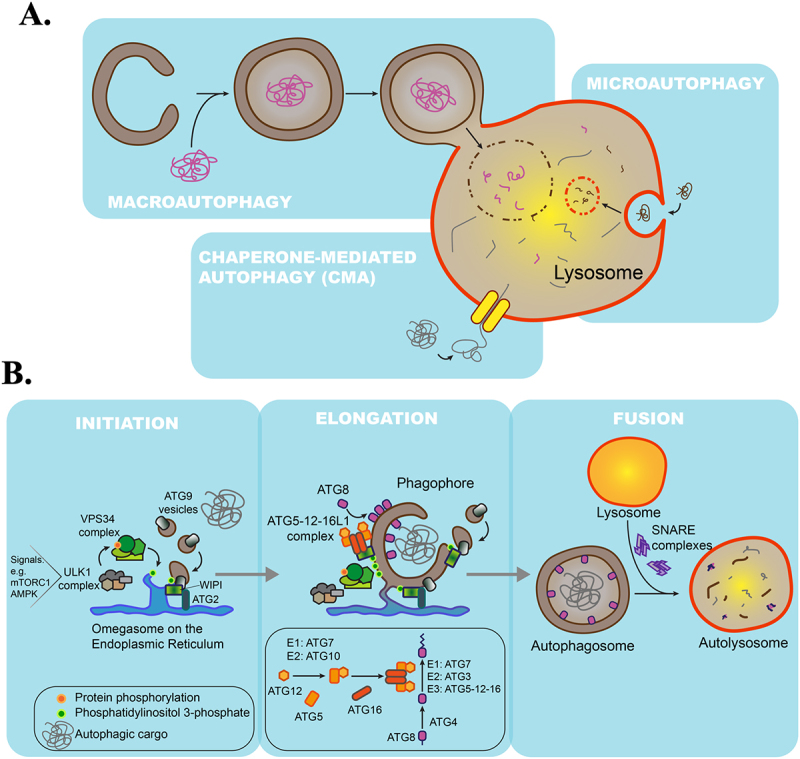
**General overview of Autophagy**. (**A**) General overview of autophagy pathways. In macroautophagy, a cup-shaped membrane, termed a phagophore, forms and grows to surround a portion of the cytosol. This seals to form an autophagosome that has a double limiting lipid bilayer. The outer membrane of the autophagosome fuses with the lysosome and delivers the inner membrane and its cytosolic components to the lysosome lumen where degradation takes place. In microautophagy, the limiting membrane of the lysosome invaginates, delivering a small internal vesicle containing cytosol to the lumen for degradation. In Chaperone-mediated autophagy, soluble cytosolic proteins are bound by chaperones and directly translocated across the lysosome membrane for degradation. For a more detailed description of the distinct autophagy pathways, the reader is referred to the following references [2-5] (**B**) Key molecular stages of macroautophagy. (Left panel) Incoming signals, such as inhibition of mTORC1, lead to activation of the ULK1 kinase complex. This in turn phosphorylates the VPS34 lipid kinase complex that produces phosphatidylinositol 3-phosphate (PI3P) at the omegasome. PI3P recruits downstream factors including the WIPIs that help coordinate incoming ATG9 vesicles, in conjunction with ATG2, to transfer lipid to the growing phagophore. (Middle panel) This elongation continues with conjugation of ATG8 proteins to the phagophore. This requires a series of ubiquitin-like conjugation reactions highlighted in the lower section of the middle panel. ATG8s help the phagophore grow and recruit additional autophagy factors including cargo. (Right panel) Once the autophagosome has formed, this is trafficked to lysosome where SNARE-mediated fusion takes place to deliver the cytosolic cargo for degradation.

Autophagy comprises a family of lysosomal degradation pathways, namely macroautophagy, microautophagy and chaperone-mediated autophagy (CMA) (Figure 2A), which perform a multitude of cellular functions [2,3]. In all these pathways, intracellular components are delivered to the lysosomal lumen, whereupon they are degraded by multiple acidic hydrolases. Macroautophagy involves the lysosomal delivery of components via a specialized organelle termed the autophagosome, which forms *de novo* to engulf multiple types of cargo ranging from protein complexes and aggregates to organelles and intracellular pathogens. Fusion of autophagosomes with lysosomes results in the formation of hybrid autolysosomes, where the autophagic cargo is degraded. Microautophagy involves direct lysosomal delivery of intracellular material, *via* invagination of the lysosomal membrane (or late endocytic compartment membranes). The resultant luminal vesicle and its contents are then recycled by lysosomal hydrolases [4]. Unlike the other pathways, the third characterized type of autophagy, CMA, is limited to degradation of soluble cytosolic proteins. CMA machinery consists of cytosolic and lysosomal chaperones that bind to proteins containing a specific pentapeptide motif. This results in unfolding and translocation of the protein into the lysosomal lumen [5].

Macroautophagy, hereafter referred to as “autophagy”, is the most understood form of autophagy and is highly conserved from yeast to man. Many excellent reviews have already been written concerning the molecular mechanisms of autophagy [for example, see [3,6]] and this will only be briefly summarized here. Autophagosome formation is initiated by four molecular protein systems. The most upstream system is the ULK1 (Unc-51 Like Autophagy Activating Kinase 1) protein kinase complex, which is thought to act as a signaling node to transduce multiple distinct upstream signals and trigger autophagosome formation. Key nutrient sensing kinases such as MTOR (mechanistic target of rapamycin) and AMPK (AMP-activated protein kinase) all phosphorylate and regulate the ULK1 complex to mediate autophagy induction. A direct target of the ULK1 complex is the second system, the VPS34 kinase complex. A main function of this lipid kinase complex is to produce the signaling lipid PI3P (phosphatidylinositol 3phosphate) at the omegasome, a special subdomain of the endoplasmic reticulum and site of the forming autophagosome. PI3P acts as a marker to further recruit factors to initiate formation of the phagophore, a membrane cisterna that grows and engulfs cytosolic components, eventually sealing to form the autophagosome. PI3P production results in the recruitment of the third autophagy system, the ubiquitinlike conjugation system. Here, the ATG (autophagy related)-8 family of proteins, which closely resemble ubiquitin (and are experimentally used as autophagosome markers), are conjugated to the lipid phosphatidylethanolamine present in the phagophore membrane through a complex set of ubiquitin-like conjugation reactions, requiring ATGs 3, 4, 5, 7, 10, 12 and 16. ATG8s, which include the LC3 (microtubule-associated protein light chain 3) and GABA type A receptor-associated proteins, are thought to enable growth of the phagophore and act as scaffolds to further recruit autophagy machinery as well as cargoes destined for degradation. The fourth system involved in autophagosome formation is the ATG9 system. ATG9 is a transmembrane protein and is key for transporting lipid required for the growth of the phagophore. Once the autophagosome has formed, conventional vesicular trafficking machinery is used to convey the organelle to the endocytic pathway, where SNARE (soluble N-ethylmaleimide-sensitive factor attachment protein receptor)-mediated fusion with late endosomes and lysosomes allows cargo degradation (See Figure 2B).

A key aspect of autophagy is the cargo that is sequestered for turnover. Early evidence showed that autophagy was a non-selective process, with cytosolic components seemingly randomly targeted for degradation. However, a wealth of evidence now shows that autophagy can also be highly selective and dependent on conditions, specific organelles or proteins can be targeted for recycling [7,8]. This is achieved by cargo recruitment of autophagy receptors, of which there are two main types. The sequestosome-like receptors [named after the archetypal member SQSTM1 (p62/Sequestosome1)] bind to ubiquitin on the cargo and then recruit the autophagy machinery. For example, during aggrephagy (the autophagy of protein aggregates) SQSTM1 binds to polyubiquitylated protein aggregates and recruits the autophagy machinery, in part by binding to LC3 proteins on the phagophore *via* its LC3-interacting regions. There are also non sequestosome-like autophagy receptors, and these can be proteins that are already present on the cargo. For example, in mitophagy (autophagy of mitochondria) one pathway involves the upregulation of the mitochondrial outer membrane protein BNIP3L (BCL2 interacting protein 3 like) and this directly interacts with LC3 family proteins through its own LC3-interacting domain (LIR) [9].

At its simplest level, autophagy is just a lysosomal degradation pathway; however, it performs various functions that are vital for cell homeostasis. Not only does it act as a recycling process to provide essential building blocks for the synthesis of new components, but it also eliminates old, toxins or dysfunctional components, which if left to persist could be damaging to the cell. As discussed below, these aspects are essential for vision and autophagy has emerged as a fundamental process in the eye.

## 3. A comparative map of Autophagy in the Vertebrate Eye

**Figure 3.**
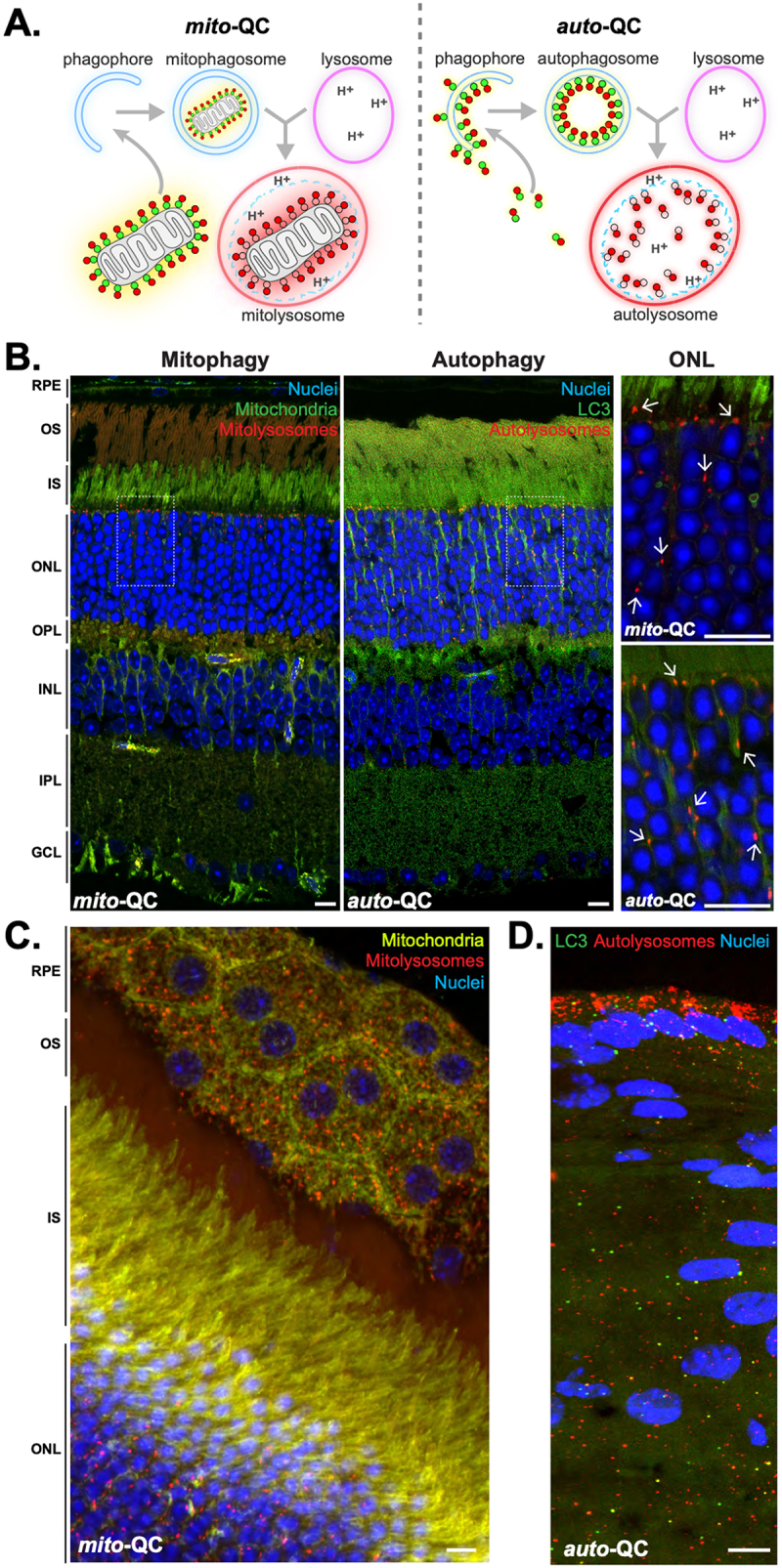
**Examples of macroautophagy in the eye from adult *mito*-QC and *auto*-QC mice**. (**A**) *Mito*QC tissue sections express a mitochondrially targeted mCherry-GFP transgene that reports on the level of mitophagy (mitochondria in cytosol fluoresce both red and green (overlaps to green-yellow color), while mitochondria delivered to mitolysosomes fluoresce red-only (due to GFP being quenched by the low pH within the autolysosome lumen). *Auto*-QC tissue sections express a related transgene with the mCherryGFP tag targeted to autophagosomes, which reports on all macroautophagy pathways (green/yellow puncta represent autophagosomes, while red represent autolysosomes). See main body of text for full description. (**B**) High levels of mitophagy in the retina. Optical section from *mito*-QC retina (right) or auto-QC retina (center). Boxed regions are shown on the right and arrows highlight examples of mitolysosomes and autolysosomes. RPE, retinal pigment epithelium; OS, outer segment; IS, inner segment; ONL, outer nuclear layer; INL; inner nuclear layer; OPL, outer plexiform layer; IPL, inner plexiform layer; GCL, ganglion cell layer. (**C**) 3D image projection of the outer retinal region from *mito*QC mice. Note hexagonal shape of RPE cells and the significant levels of mitophagy. (**D**) Z-projection of lens optical slices from the equatorial region of auto-QC mice. Note the high number of autophagosomes (green) and autolysosomes (red). Scale bars, 10 *μ*m. Images are derived from [15] and reproduced under the terms of the Creative Commons Attribution License.

To study autophagy in the eye, it is critical to be able to visualize the process. While it is now relatively easy to study autophagy in isolated cells grown in culture, it becomes challenging in complex organs such as the eye, which consist of multiple cell types with intricate three-dimensional architectures. Though there are now many technical approaches to study autophagy, both in vitro and in vivo [10], the generation of fluorescent autophagy reporter animals has greatly facilitated this process. With respect to mammalian systems, the transgenic GFP-LC3 mouse was one of the initial models used to study autophagy in the eye [11,12]. Following this, additional reporter models have allowed a more in-depth characterization of autophagy within the eye. Recently we generated two very similar transgenic mouse models that express a tandem mCherry-GFP tag attached either to LC3, enabling visualization of general autophagic flux, or attached to the mitochondrial outer membrane, enabling visualization of mitophagy [13,14] (Figure 3A). The tandem mCherry-GFP tag acts as a pH-sensitive autophagic flux sensor as when the tag is in the cytosol, both fluoresce. However, upon autophagy the tag will be delivered to lysosomes where the acidic luminal environment is sufficient to quench the GFP signal, but not that of mCherry, resulting in an easily detectable color change. As these mice were generated in the same way under the same conditions, they have allowed a comparative assessment of the specificity of autophagy, at least in terms of mitophagy, within the eye [15]. Firstly, these mice have revealed that autophagy in general is a pervasive process within the eye, with significant but varying levels observed in every section of the eye analyzed. Secondly, they have shown that the level of mitochondrial autophagy is simply not proportional to the level of general autophagy present, implying cell-specific autophagy signaling. This is highlighted when comparing two distinct tissues: the lens and the retina. High levels of autophagy are apparent in the lens, as evidenced by the large number of autophagosomes and autolysosomes present. This is most obvious in the lens epithelial cells. In contrast, in the same epithelial region, the mitophagy reporter mouse shows very little evidence of mitophagy, even though the reporter clearly marks mitochondria. As will be discussed below, mitophagy in the lens may well be limited in terms of organelle clearance. In contrast to the lens, mitophagy clearly occurs in the retina. Both reporter models show comparatively high levels of autophagy and mitophagy in the outer nuclear layer, with both rod and cone photoreceptor cells demonstrating that a significant proportion of autophagy here is due to mitochondrial turnover. Taken together, these data not only highlight the specificity of the two reporter systems but show that although autophagy is abundant in multiple cell types of the eye, it is likely performing distinct and specific functions, at least under the basal conditions of this study (see Figure 3B-D for example images). We will now go on to discuss the spatio-temporal nature of ocular autophagy in more detail.

## 4. Autophagy in the developing eye

Autophagy plays a key role during development as evidenced by the embryonic lethality of several autophagy knockout animals like *Becn1^−/−^* (*Beclin 1*) and *Ambra1^−/−^* (autophagy and beclin 1 regulator 1). These findings have increased research interest in the developmental role of autophagy. Autophagy proteins are expressed at high levels during eye development [16], and a great number of red dots indicative of autophagy and mitophagy are observed in the developing eye and lens at E16.5 [15]. Historically the initial reports showed that autophagy was associated to cell death during embryonic development - as autophagosomes were often observed in dying cells in developing tissues. These findings were used to “define” autophagic cell death based on morphological criteria. In the retina, Linden and coworkers began to study the pathways implicated in programmed cell death during postnatal retinal development and observed autophagy-dependent cell death in retinas cultured ex vivo [17]. Later studies performed in chick retinas demonstrated the metabolic role of autophagy in preserving ATP levels required for the elimination of apoptotic cells during development [16,18], a phenomenon that was also observed during cavitation of embryonic bodies [19]. The existence of autophagy activity during eye development was further corroborated in a later study demonstrating that cell death during hyaloid vessel regression is dependent on autophagy [20].

*Ambra1* knockout in zebrafish results in reduced eye size, smaller otic vesicles, and embryonic lethality [21]. *Atg5*-deficient animals display increased cell death in the retinal neuroepithelium and display important alterations in the developing RGCs- the first retinal neurons to be differentiated- with axonal defects and increased cell death [19,22]. More importantly this autophagy activity is associated to the elimination of mitochondria via mitophagy as *Atg5*-deficient retinas at E15.5 display an accumulation of mitochondria, a phenotype that is also observed in the mitophagy *BNIP3L* (BCL2 interacting protein 3 like)-knock out animals [22]. In this setting mitophagy is upregulated as a cellular response to hypoxia which increases the mRNA expression of BNIP3L, a target of the hypoxia-responsive transcription factor HIF1a. The hypoxic response is associated with a peak in glycolysis at these embryonic stages that is essential for RGC differentiation. Increasing glycolysis by blocking the entry of pyruvate into the mitochondria resulted in increased RGC number while reducing glycolysis with 2-dexyglucose reduced the number of newly differentiated RGCs. Interestingly, increased glycolysis did not alter mitochondrial number, indicating that the metabolic change essential for cell differentiation occurs downstream of mitophagy [22]. These data show that autophagy and mitophagy are also used by cells to regulate cellular metabolism and that mitophagy by eliminating mitochondria can control the balance glycolysis-oxidative phosphorylation. More importantly this response seems to be universal, and macrophages also use mitophagy to perform a glycolysis shift during their inflammatory response. Why developing RGCs change metabolism during neurogenesis remains to be fully explained, but it could be a way for example to eliminate damaged mitochondria resulting from reactive oxygen species (ROS) production in proliferative neuroblasts. Taken together, these findings demonstrated that autophagy plays an active role in the physiological retina, participating in programmed cell death from early developmental stages and modulating metabolisms and cell differentiation during retinal neurogenesis.

## 5. Corneal resiliency through autophagy regulated homeostasis

The cornea is the outermost refractive element of the eye made up of a multilayered epithelium, stroma, and a single-layered endothelium. The tear wettened corneal epithelium is the barrier to the external environment. Its turnover rate is every 7-10 days [23] [24], much more rapid than the epidermis of skin at 40 - 56 days [25]. The stroma is a relatively thick region of regularly arranged collagen fibers that minimize light scatter while providing structural integrity [26]. The innermost endothelium is a single layer of non-proliferative cells responsible for corneal deturgescence and stromal nutrient supply from the aqueous humor [27]. Regeneration of the corneal epithelium and transparency of the corneal layers are dependent on autophagy. Here we briefly introduce how autophagy contributes to corneal homeostasis and when dysregulated to vision impairing stromal and endothelial dystrophies. For a more comprehensive description of autophagy in the normal and diseased cornea [28].

### 5.1 Role of Autophagy in corneal epithelial regeneration

Corneal epithelial turnover is dependent on the proliferation of limbal stem cells located at the junction of the cornea with surrounding conjunctiva. MIR103-107 micro-RNA stimulation of end-stage autophagy is essential for limbal stem cell regenerative activity through diacylglycerol, protein kinase C and cyclindependent kinase 5 signaling. It also coordinately represses macropinocytosis, to together regulate limbal stem cell homeostasis [29]. Limbal stem cells in turn give rise to transit-amplifying cells that migrate to and with differentiation replenish the peripheral and central cornea [30]. Subsequent single-cell RNA sequencing of autophagy defective *becn1^+/-^* mice display a 50% loss of limbal stem and transit amplifying cells together with decreased expression of proliferation-associated genes such as *Mki67* (antigen identified by monoclonal antibody Ki 67) and *Lrig1* (leucine-rich repeats and immunoglobulin-like domains 1) [31].

### 5.2 Autophagy in corneal epithelial diseases

#### 5.2.1 Autophagy and Dry Eye

Dry eye is a common tear deficiency disease associated with altered visual acuity, corneal epithelial damage, corneal nerve and tear depletion and accompanying inflammation. Lacritin, a tear, plasma and CSF protein discovered out of an unbiased biochemical screen to address dry eye promotes basal tearing and restores homeostasis in dry eye mouse models [32-35]. Patients suffering from dry eye are selectively lacritin monomer deficien [36-38]. Use of mCherry-GFP-LC3 transfected human corneal epithelial cells stressed with inflammatory cytokines or overexpressing huntingtin mutant Htt103Q reveals that lacritin transiently accelerates autophagic flux thereby restoring oxidative phosphorylation through enhanced mitochondrial fusion. Signaling is mediated by lacritin dependent acetylation of FOXO3, as a novel ATG101 ligand, and involves kinetically slower lacritin dependent coupling of stress-acetylated FOXO1 with ATG7. Lacritin C-terminal synthetic peptide ‘N-94/C-6’ (‘Lacripep’) is equally active on aire^−/−^ dry eye mice [33], and in initial humans studies [phase I/II trial (NCT03226444); [39]].

#### 5.2.2 Autophagy in Keratoconus

Corneal thinning and astigmatism are the main features of keratoconus, the leading cause of corneal transplantation in the United States. Analysis of keratoconus patient samples show decreased levels of autophagosome marker LC3-II and LAMP1 (lysosomal membrane protein 1) and increased levels of SQSTM1 in the diseased corneal Grades I and III cone-specific epithelium compared to the matched control peripheral region [40]. These results suggest that dysfunctional autophagy may be present in keratoconus. Two studies have identified oxidative stress as the cause of autophagy dysfunction in cultured corneal epithelial cells [40,41]. Interestingly, Shinde *et al*. found diminished NRF2 (nuclear factor erythroid 2–related factor 2) anti-oxidant response signaling pathways in keratoconus patient samples [42].

#### 5.2.3 Autophagy in Microbial Infections

Pathogenic fungi, viruses and bacteria modulate corneal epithelial autophagy using different approaches - some as autophagic antagonists and others as agonists, or some as both [28]. One example of the latter is Herpes Simplex Virus -1 (HSV-1), a leading cause of blindness. HSV-1 surface glycoproteins gB, gC and gD target cell surface heparan sulfate on proteoglycans to initiate membrane fusion [43], entry and subsequent autophagy via STING1 (stimulator of interferon response CGAMP interactor 1) [44]. This is followed by BECN1 ligation by HSV-1 ICP34.5 to antagonize autophagy and thereby facilitate HSV-1 expansion [45]. HSV-1 also triggers the formation of ‘autophagic clusters’ in the ophthalmic branch of the trigeminal ganglia of cells adjacent to but not positive for HSV-1 antigen, a process interpreted as a neuronal anti-viral response linked to the establishment of latency [46]. Another example is *Pseudomonas aeruginosa* that injects exoenzyme effectors ExoS and ExoU into host cells via its type III secretion system. ExoS’ C-terminal ADP-ribosylation domain [47,48] partially inhibits host cell PIK3C3 (phosphatidylinositol 3-kinase catalytic subunit type 3, also known as VPS34) necessary for the autophagy initiating ATG14-beclin 1 complex [47]. In contrast, ExoU embeds in mitochondiral membranes to provoke mitophagy and oxidative stress as a ubiquitin-activated phospholipase A2. An example each of an autophagic antagonist and agonist are respectively Rubella virus (per LC3B-II blotting in the absence or presence of bafilomycin, an effect reversed by rapamycin) [49] and *S. marcescens’* prodigiosin (proapoptotic red pigment) and ShlA (pore forming toxin) [28,50].

### 5.3 Autophagy and corneal stromal diseases

While very little is known about the role of autophagy in the homeostasis of the corneal stroma, its function is well-studied in disease. Epithelial-stromal dystrophies are a group of conditions that arise due to mutations in the *TGFBI* (transforming growth factor beta-induced-1) gene leading to stromal accumulation of mutant TGFβ1 protein associated with impaired autophagic clearance [51,52]. Rescue was achieved in a cell model by over-expression of lysosomal regulator TFEB (transcription factor-EB) [52]. Macular corneal dystrophy is a rare disease distinguished by stromal accumulation of keratan sulfate precipitates [53]. Patient keratocytes reveal a reduction in the autophagy-lysosomal pathway and activation of pyroptosis mediated cell-death [54].

### 5.4 Autophagy in the corneal endothelial dystrophies

Corneal endothelial dystrophies are bilateral, non-inflammatory diseases that arise due to malfunctions or loss of corneal endothelium. Endothelial dystrophies are linked to or caused by mutation of *SLC4A11* (solute carrier family 4 member 11), *COL8A2* (collagen type VIII alpha 2 chain), *LOXHD1* (lipoxygenase homology PLAT domains 1), *AGBL1* (AGBL carboxypeptidase 1), *COL17A1* (collagen type XVII alpha 1 chain) or *TCF4* (transcription factor 4). Dysregulation of autophagy appears to play a role in the pathogenesis of several endothelial dystrophies. A late autophagic block in SLC4A11 null cells was demonstrated by a strong GFP signal in GFP-LC3-RFP-LC3Δ transfected cells together with a deficiency of the multisubunit vacuoloar ATPase and of CTSB and decreased nuclear localization of TFEB [55]. Quenching mitochondrial oxidative stress with MitoQ reduced autophagy dysfunction and reduced corneal endothelial pathology [55]. *Col8a2*^Q455K/Q455K^ transgenic mice mimic human corneal endothelial dystrophy including the gradual depletion of corneal endothelial cell density. Feeding lithium carbonateenriched chow to stimulate autophagy partially restored corneal endothelial cell density [56]. In contradistinction, Fuchs corneal endothelial dystrophy from mutated *TCF4* is characterized by decreased mitochondrial mass coupled with activated mitophagy. This could be partially restored by blocking autophagic flux with bafilomycin [57]. In *Col8a2*^Q455K/Q455K^ and *Col8a2*^L450^^W/L450^^W^ mouse models of Fuchs corneal endothelial dystrophy, increased expression of LC3-I protein and DRAM1 (DNA Damage Regulated Autophagy Modulator 1) mRNA - the latter required for p53 triggered autophagy and apoptosis [58].

## 6. Autophagy-dependent and independent intracellular degradation systems in the lens

### 6.1 Structure and function of the lens: A need for a powerful degradative system

**Figure 4.**
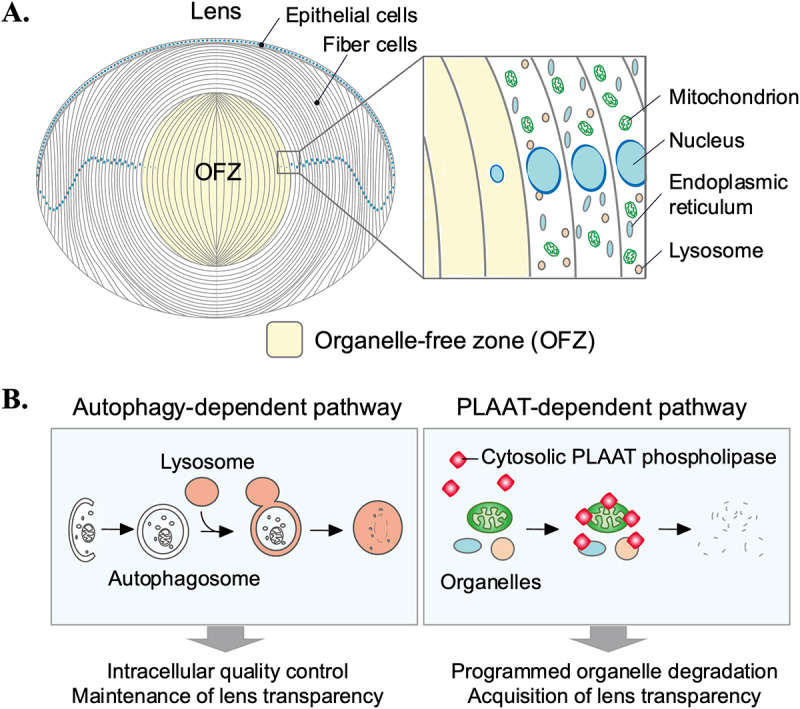
**Functions of autophagy-dependent and independent mechanisms in the lens. (A)** Schematic presentation of the mammalian lens and the process of programmed organelle degradation in the lens. **(B)** Functions of autophagy-dependent and PLAAT-dependent pathways in the lens.

The ocular lens is an avascular and transparent tissue composed of epithelial cells and fiber cells [59,60] (Figure 4A). Lens epithelial cells, which cover the anterior surface of the lens, differentiate into fiber cells that make up the bulk of the lens. The epithelial-to-fiber differentiation is accompanied by a series of spatiotemporally organized processes, including cell elongation, production of crystallins necessary for transparency and refractive power of the lens, and ultimate degradation of nuclei and other membranebound organelles [59,61]. The function of the lens is to transmit light and sharply focus it onto the retina. To achieve this, the lens must be transparent and free of light-scattering substances.

### 6.2 Mechanisms of programmed organelle degradation in the lens

One of the most remarkable features of lens fiber cell differentiation is the programmed degradation of all membrane-bound organelles, including the nuclei, endoplasmic reticulum (ER), and mitochondria [60,61] (Figure 4A). It is an evolutionarily conserved phenomenon that occurs from the center to the periphery of the lens, forming the organelle-free zone (OFZ) [60-65]. A longstanding unanswered question in cell biology has been how membrane-bound organelles are degraded in the lens. Previous studies have shown that degradation of nuclear DNA in the lens is mediated by lysosomal DNASE2B (deoxyribonuclease 2 beta) in mice [66-68] and Dnase1l1 (deoxyribonuclease-1-like 1) in zebrafish [69,70], and depends on several other regulators of lens development and differentiation [59,71-73]. However, the degradation mechanisms of other membrane-bound organelles, such as mitochondria and the endoplasmic reticulum (ER), have long been unknown. It appears to be at least partially dependent on HSF4 (heat shock transcription factor 4) [74-76], but the executor of organelle degradation has not been identified. A previous study proposed that 15-lipoxygenase is involved in this process [77]. However, contrary to the report, its expression does not increase during lens fiber cell differentiation [67] and its function in the lens has not been verified *in vivo* [78,79].

The contribution of autophagy in lens organelle degradation has been investigated, but it is currently under debate [61,80]. The contribution of autophagy in lens organelle degradation has been investigated, but it is currently under debate [61,80]. Previous studies using electron microscopy reported the presence of autophagic vesicles containing mitochondria in lens epithelial cells and fiber cells during early stages of differentiation [81]. The involvement of the mitophagy receptor BNIP3L and hypoxia in the elimination of mitochondria, the endoplasmic reticulum and the Golgi during OFZ formation was also suggested [8184]. Moreover, chemical inhibition of the JNK (c-Jun N-terminal kinase), MTORC1 (mTOR complex 1), or PtIns3K (phosphoinositide 3-kinase) signaling pathways stimulates premature organelle degradation by autophagy in an *ex vivo* culture system of chick embryo lenses [82-85], suggesting that lens organelles can be degraded by enhanced autophagy or other mechanisms. However, multiple lines of *in vivo* genetic evidence indicate that autophagy is not required for organelle degradation during lens fiber cell differentiation under physiological conditions. For example, organelle degradation normally occurs in mice lacking the essential autophagy genes *Atg5* [11,86], *Pik3c3* [11], or *Rb1cc1* (RB1 inducible coiledcoil 1, also known as *Fip200*) and in zebrafish lacking *rb1cc1* [87]. Furthermore, unbiased studies using mitophagy reporter mice (*mito*-QC mice) show no evidence of upregulation of mitophagy in lens fiber cells [15].

Recently, it was found that cytosolic PLAAT (phospholipase A and acyltransferase)-family phospholipases are essential for the degradation of lens organelles such as the ER, mitochondria, and lysosomes [87,88] (Figure 4B). PLAAT is a phospholipase A that is highly conserved among vertebrates and has activity toward a variety of glycerophospholipids [89,90]. In the lens, zebrafish Plaat1 (also known as Hrasls) and mouse PLAAT3 (also known as HRASLS3, PLA2G16, H-rev107, and AdPLA) are highly expressed in fiber cells and are essential for organelle degradation [87]. PLAAT has a transmembrane domain at the C-terminus, but PLAAT is primarily present in the cytosol. However, upon membrane damage (e.g., pore formation), PLAAT can localize to organelles when their membranes are mildly damaged and induce complete degradation of these organelles [87]. Damage to the lysosomal membrane allows leakage of dNase into the cytosol and subsequent degradation of nuclear DNA. The upstream and downstream events of PLAAT-mediated membrane rupture are not known. The initial membrane damage depends on HSF4 [87]. HSF4-dependent upregulation of proteins (e.g., high levels of crystallins) may affect membrane homeostasis and induce membrane damage. Which factors are transcriptionally activated by HSF4 need to be identified. HSF4 is expressed and activated in the central region of the lens [91-94]. This activation pattern may explain why organelle degradation spreads from the center to the periphery. Zebrafish deficient in *plaat1* and mice deficient in *Plaat3* develop cataracts, indicating that lens organelle degradation is essential for acquiring lens transparency. These findings propose an evolutionarily conserved autophagy-independent mechanism for lens organelle degradation, in which cytosolic PLAAT localizes to organelles to induce membrane degradation (Figure 4B). This system is distinct from autophagy and microautophagy, in which organelles are degraded within lysosomes. To degrade all organelles in lens cells, the mechanism using a cytosolic factor may be more efficient than autophagy because there is no need for autophagosomes and lysosomes, respectively, to engulf organelles one by one. It will be important to investigate the relationship of PLAAT with the lysosomal and the ubiquitinproteasome degradation systems. Also, since PLAAT is expressed in tissues other than the lens, it would be interesting to elucidate the role of PLAAT in organelle degradation in other tissues.

### 6.3 Roles of autophagy in lens physiology and suppression of cataracts

Like neurons, lens fiber cells are quiescent, and all lens fiber cells are retained in the lens throughout life [59,60]. Therefore, the intracellular quality of lens fiber cells must be tightly controlled in order to maintain lens transparency. The rate and speed of lens fiber cell differentiation decrease with age; the process from final mitosis to denucleation in rodents requires only about a week if initiated during the embryonic period, whereas it takes about 9 months after 5 months of age [95,96]. Therefore, efficient intracellular quality control is needed, especially in lens fiber cells of the adult lens.

Several studies have suggested a role for autophagy in maintaining lens homeostasis and transparency (Figure 4B). First, studies using mice expressing GFP-LC3 [11,12, 86] or mCherry-GFP-LC3 [15] and zebrafish expressing GFP-LC3-RFP-LC3ΔG [97] have revealed that autophagy is constitutively activated in both lens epithelial and fiber cells. Electron microscopy has also demonstrated the presence of autophagosomes in lens fiber cells [81]. Next, lens-specific *Atg5*-deficient mice show accumulation of autophagic substrates such as polyubiquitinated proteins and SQSTM1 and eventually develop ageassociated cataracts with increased levels of oxidized proteins and insoluble crystallins [11]. In addition, the importance of endolysosomes, which are required for both autophagy and endocytosis, in lens development and the suppression of cataracts has also been reported. For example, lens-specific depletion of PIK3C3, which produces phosphatidylinositol 3-phosphate (PtdIns(3)P) and is essential for autophagy, endocytosis and multivesicular body formation [98,99], shows accumulation of autophagic substrates and enlarged endolysosomes and congenital cataracts [11]. Thus, autophagy is required for intracellular quality control.

### 6.4 Roles of autophagy in human cataracts

Cataractogenesis is a multifactorial process, and aggregation of misfolded crystallins is a common feature of several types of human cataracts [100,101]. αA-crystallin and αB-crystallin account for about 30% of the protein content of the lens and are responsible for maintaining the solubility of other lensproteins such as β-crystallin and γ-crystallin [102,103]. Continuous damage to these proteins can lead to age-related cataracts with aggregation of crystallin proteins [102,103]. The αB-crystallin knock-in mouse model encoding the R120G mutation, which causes autosomal dominant hereditary myopathy and cataracts in humans [104], suggests a defect in autophagy [105,106]. In this model, cataracts are associated with the accumulation of SQSTM1-positive aggregates due to insufficient autophagy and increased αB-crystallin aggregation [105,106]. Similar autophagy defects have been reported in another knock-in mouse model of the αA-crystallin R49C mutation [107], which is responsible for autosomal dominant hereditary cataracts [107,108]. Similarly, mice lacking βA3/A1-crystallin, which is mutated in autosomal dominant cataract in humans [109], also develop cataracts with the accumulation of autophagic substrates [110]. Thus, mutations in the crystallin gene can be associated with reduced autophagy, though its mechanism remains unclear.

Several other studies also suggest associations of the suppression of autophagy and the endolysosomal systems with human hereditary cataracts with mutations in non-crystallin genes. Mutations in *FYCO1* (FYVE and coiled-coil domain autophagy adaptor 1), a gene encoding a Rab7 effector that binds to LC3 and PtdIns(3)P and regulates the transport of autophagosomes along with microtubules, are associated with autosomal recessive congenital cataracts [111-113]. Mice deficient in *Fyco1* exhibit normal lens organelle degradation [114], but develop age-associated cataracts with the accumulation of SQSTM1, insoluble crystallin [114], and some organelles in differentiating lens fiber cells around the OFZ [115]. Thus, FYCO1 is not required for lens organelle degradation (i.e., formation of the OFZ), but is important for intracellular quality control.

Mutations in *CHMP4B* (charged multivesicular body protein 4B), a gene encoding a key component of the ESCRT (endosomal sorting complex required for transport), are associated with autosomal dominant cataracts [116]. CHMP4B plays important roles in autophagosome formation, multivesicular body formation, nuclear envelope reformation, and cytokinesis [117-120]. Lens-specific *Chmp4b*-deficient mice develop congenital cataracts with severe cellular damages [121], probably due to various dysfunctions in CHMP4B-related pathways.

Mutations in *EPG5* (ectopic P-granules autophagy protein 5 homolog) gene results in the recessive multisystem disorder Vici syndrome characterized by congenital cataract, callosal agenesis, combined immunodeficiency, cardiomyopathy, and hypopigmentation [122]. EPG5 is a Rab7 effector involved in autophagosome maturation (fusion of autophagosomes with endolysosomes) [123,124]. However, *Epg5*deficient mice do not mimic all the symptoms of Vici syndrome; some features of the disease, such as hypopigmentation, facial dysmorphism, and cataract, are absent [125,126]. The reasons for the phenotypic differences between humans and mice need to be clarified by further studies.

Mutations in *TBC1D20* (TBC1 domain family member 20), a gene associated with the autosomal recessive disorder Warburg Micro syndrome 4 characterized by congenital abnormalities in the eye (including cataracts), brain, and genital organs [127], show impaired autophagy [128]. A similar phenotype has been reported in patients with mutations in *TDRD7* (tudor domain containing 7), a gene associated a syndrome combining congenital cataracts and non-obstructive azoospermia [129,130]. TDRD7 is a component of cytoplasmic RNA granules and is suggested to GTPase-activating protein (GAP) for Rab1 [129]. Mice lacking *Tbc1d20* or *Tdrd7* develop cataracts with disrupted autophagic flux and delayed differentiation in lens fiber cells [128,129]. TBC1D20 is a GAP for Rab1 [131], which is considered a housekeeping Rab required for the secretory pathway, maintenance of the Golgi structure, and regulation of autophagy [132], suggesting complex pathogenesis underlying these diseases.

Mutations in *RRAGA* (Ras related GTP binding A), the gene encoding the RagA GTPase that regulates mTORC1, are associated with autosomal dominant cataracts [133]. The mutations activate mTORC1 signaling, including increased RagA relocation to the lysosomes, downregulation of autophagy, and decreased cell growth [133]. Since mTORC1 has diverse roles, it remained unknown whether the cataract formation is caused by impaired autophagy.

Mutations in *PIKFYVE* (phosphoinositide kinase, FYVE-type zinc finger containing), a gene encoding a protein that produces PtdIns(3,5)P_2_ and plays important roles in the endocytosis and autophagy [134], are associated with autosomal dominant cataracts [135] and fleck corneal dystrophy [136]. Zebrafish deficient in *pikfyve* develop congenital cataracts with enlarged endolysosomes, which are likely amphisomes because they are positive for Rab7 and LC3. Suppression of the acidification of endolysosomes using bafilomycin A_1_ alleviates these phenotypes, suggesting that the vacuolation rather than impaired autophagy may be the primary cause of the cataract.

Mutations in *GJA8* (gap junction protein alpha 8, also known as connexin 50) lead to autosomal recessive cataracts of various different phenotypes [137]. Zebrafish deficient in *gja8b*, a gene encoding the homolog of mammalian GJA8, develop cataracts with impaired autophagy and terminal lens fiber differentiation, including degradation of the ER, nuclei, and aquaporin 0 [138]. These phenotypes, including cataracts, can be relieved by enhanced autophagy using rapamycin, an mTORC1 inhibitor [138]. It is expected that the mechanism by which GJA8 deficiency causes autophagy suppression and differentiation defects will be clarified in the future.

In summary, while the relationship between autophagy suppression and cataracts has been suggested and supported by animal models, the actual mechanism for cataractogenesis is not well understood. Whether activation of autophagy can improve cataracts is also currently unknown. Paradoxically, rapamycin, a typical autophagy-inducing drug, causes cataracts as a side effect [139]. Therefore, it is necessary to develop specific autophagy-regulating drugs in the future.

## 7. Autophagy in the Conventional Outflow Pathway: implications in aqueous humor homeostasis

### 7.1 Introduction to the conventional outflow pathway

The conventional outflow pathway, composed of the trabecular meshwork (TM) and the Schlemm’s canal (SC), is a mechanosensitive tissue that is located in the anterior segment of the eye, at the intersection between the cornea and the sclera. The main role of this tissue is to regulate the pressure inside the eye (intraocular pressure, IOP). Functional failure of the conventional outflow pathway, normally ocurring with aging and in disease causes elevated IOP, namely ocular hypertension (OHT) and increases the risk for developing glaucoma [140]. As discussed in [141], a particularity of the cells in the outflow pathway is that they are continuously subjected to a variety of physiological stresses as part of their normal metabolism (oxidative stress, mechanical stress and phagocytic stress). Thus, it is crucial that these cells account with efficient protective and repair mechanisms. A number of studies, summarized here, have investigated the contribution of autophagy in outflow pathway physiology and pathophysiology.

### 7.2 Autophagy and Mechanical Forces

In contrast to what was initially thought, live recording of IOP in humans and monkeys have shown that IOP is not fixed, but it is constantly subjected to fluctuations resulting from eye movement (up to 10 mmHg) and ocular pulse (2-3 mmHg) [142]. Cells in the outflow pathway sense these fluctuations in IOP as mechanical forces in the form of stretching/relaxation and react to them by eliciting an array of responses, which are thought to be critical in maintaining IOP homeostasis [143].

**Figure 5.**
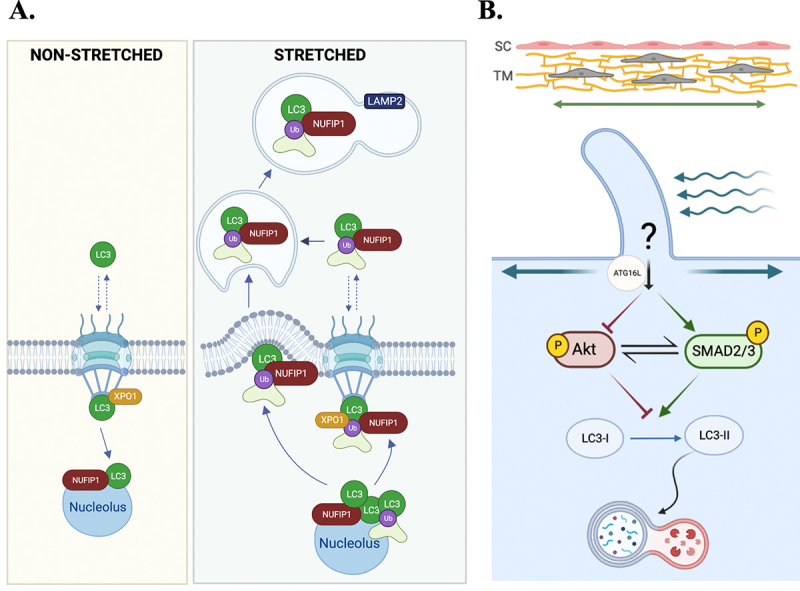
**Schematic representations of the roles of stretch-induced autophagy in the outflow pathway. (A)** Nuclear translocation of LC3 upon mechanical stretch in TM cells. Under non-stretched control conditions, LC3 is located primarily in the cytosol, shuttling in-and-out of the nucleus. LC3 enters the nucleus by passive diffusion and exits in an XPO1 active-dependent manner. Mechanical stress triggers activation of autophagy and translocation of LC3 to the nucleus. In the nucleus, LC3 is localized in the nucleolus interacting with the autophagy receptor NUFIP1. LC3-NUFIP1 complex is proposed to recognize stretch-induced damaged nuclear proteins and facilitate their export, either via active nuclear transport or nuclear envelope budding, for autophagic degradation in the cytosol. **(B)** PC-mediated stretched-induced autophagy. PC senses mechanical forces and activate autophagy. Autophagy activation is mediated by the recruitment of ATG16L at the PC basal and is regulated by a cross-talk between AKT1 and SMAD2/3 signaling pathways, acting AKT1 as an inhibitor and SMAD2/3 as an activator.

By using the Flexcell tension system, Porter et al. [144] reported autophagy as one of the responses triggered in TM cells in response to mechanical stress. Their study showed the quick activation of autophagy (within 30 minutes) in primary cultures of porcine and human TM cells subjected to either cyclic or static biaxial strain, which was characterized by increased LC3-II levels and LC3 puncta. Autophagy flux, monitored via tfLC3 assay, was not affected. Activation of autophagy in outflow pathway cells was also observed in organ culture of porcine and human eyes subjected to high pressure [144,145]. Interestingly, application of mechanical forces triggered the nuclear translocation of the autophagy marker, LC3 [145]. LC3 is a cytosolic protein that lacks nuclear import or export signals. Entry of LC3 to the nucleus was determined to occur by passive diffusion, whereas its export was mediated through active transport, most likely by interactions with proteins containing nuclear export signals. In the nucleus, LC3 was found to localize in the nucleolus, interacting with the autophagy receptor NUFIP1 (nuclear FMR1 interacting protein 1) - a nucleolar protein previously identified as a receptor for starvation-induced ribophagy [146]- and SQSTM1. Moreover, mechanical stress caused the redistribution of NUFIP1 from the nucleus to autolysosomes. These observations suggest that LC3-NUFIP1 might act as a survey complex that recognize stretch damaged nuclear proteins and deliver them for autophagic degradation (Figure 5A).

A very recent study by Shim et al. [147] identified primary cilium as the mechanosensor for stretchinduced autophagy in TM cells. Chemical disruption of primary cilia completely abolished the increase in LC3-II levels in cells subjected to mechanical forces. As in the case of starvation- and fluid flow-induced autophagy, ATG16L was observed at the basal cilium in the stretched cultures, further confirming recruitment of ATG16L as a hallmark for primary cilia-dependent autophagy [148]. The signaling pathway mediating primary cilia-dependent stretch-induced autophagy was additionally investigated. Activation of autophagy by mechanical stretch in TM cells was deemed to be mTOR-independent [144] and mediated by a newly identified cross-regulatory talk between AKT1 (AKT serine/threonine kinase 1) (AKT1) and noncanonical SMAD2/3 (suppressor of mothers against decapentaplegic 2/3) signaling controlled by primary cilium, in which AKT1 functions as a negative regulator, whereas SMAD2/3 functions as an activator [147]. Although the exact mechanisms need still to be elucidated, primary cilium was found to directly regulate AKT1 activation to control the induction of autophagy (Figure 5B).

The physiological relevance of stretch-induce autophagy was demonstrated in the organ perfused anterior segment model [147]. The investigators found that chemical removal of primary cilia disrupted the homeostatic IOP compensatory response and prevented the increase in LC3-II protein levels in response to elevated pressure challenge, strongly supporting a role of primary cilia-mediated autophagy in regulating IOP homeostasis.

### 7.3 Autophagy as a regulator of TGFβ-induced fibrosis in TM cells

Fibrosis has been implicated in the pathophysiology of OHT and glaucoma. Remarkably, transcriptome and functional network analyses identified TGFβ-induced endothelial to mesenchymal transition as one of the top biological pathways affected in TM cells deficient in autophagy [149]. The investigators found the dysregulated expression of several genes participant of this pathway, including *TGFβ2, ACTA* (⍺smooth muscle actin, also known as SMA), *BAMBI* (BMP and activin membrane-bound inhibitor), *PAI1* (plasminogen activator inhibitor 1) in TM cells with co-silenced expression of *atg5* and *atg7*. Of particular interest is the upregulated expression and secretion of TGFβ2 in autophagy-deficient cells, as TGFβ2 is present at higher concentrations in the AH from glaucoma patients [150]. Intriguingly, despite being TGFβ2 a transcriptional activator of *ACTA*, the investigators found that the levels of ACTA were decreased in autophagy-deficient cells. Similarly, genetic or pharmacological inhibition of autophagy also blocked the upregulation of the fibrotic markers, collagen I and fibronectin upon TGFβ1 or TGFβ2 treatment. Additionally, autophagy-deficient cells showed diminished levels of phosphorylated Smad2/3 (pSMAD2/3) in response to TGFβ, which were rescued by silencing the expression of *BAMBI*. BAMBI is an antagonist of TGFβ signaling also upregulated in autophagy-deficient TM cells. Interestingly, BAMBI is predominantly degraded through the autophagy lysosomal pathway [151]. Based on these findings, the authors postulated a potential role of autophagy in regulating fibrosis by abrogating TGFβ/Smad signaling in TM cells via selective autophagic degradation of BAMBI.

### 7.4 Autophagy in the Ageing Outflow Pathway

Resistance to aqueous humor (AH) outflow through the conventional outflow pathway increases with aging. Usually, this increase in outflow resistance is partially compensated by an also age-related decline in AH production, preventing IOP from becoming substantially elevated with age. However, this is not true in ocular hypertensive and glaucoma patients [152]. How aging of the outflow pathway predisposes to disease is currently not known. One potential factor is dysregulated autophagy. Pulliero et al. [153] investigated autophagy in dissected TM tissue from human cadaver eyes and found a parallel increase in LC3-II, oxidative damage, and age. Since the expression of SQSTM1 was lower, it was concluded that autophagy is activated in the aging TM. Similar increased in LC3-II, with lower SQSTM1 levels were observed in the iridocorneal angle region of old (18 m.o) mice compared to young (4 m.o) ones [154]. LAMP1 expression levels were additionally monitored in this study and found unchanged with aging, which was interpreted as a potential indication of diminished autophagy flux. Interestingly, a correlation between LC3 ratio and IOP was observed.

The effect of aging on autophagy function in TM cells was further investigated *in vitro* using a normobaric hyperoxic model of aging [155]. This model consists of culturing confluent monolayers of TM cells under 40% O_2_ conditions while keeping the controls under physiological 5% O_2_ [156]. Autophagy was found to be activated in the cultures grown under a hyperoxic environment [155]. Activation of autophagy was MTOR-dependent and involved the nuclear translocation of TFEB. Accordingly, oxidized cultures showed the upregulated expression of autophagy and lysosomal genes. However, despite such transcriptional activation and increased protein content of the autophagic and lysosomal machinery, lysosomal degradation was not enhanced in the stressed cultures. Indeed, cells grown at 40% O_2_ conditions showed lower cathepsin activities. Defective proteolytic maturation of the inactive pro-forms in enrichedlysosomal fractions was also noted. Moreover, lysosomal pH was monitored and found to be less acidic in the stressed cultures compare to those grown under physiological conditions. The cause/s underlying basification of lysosomes in TM cells under chronic oxidative stress conditions were not investigated, but it is likely mediated by the reported decreased in v-ATPase activity in aging cells [157].

In the same study, authors investigated the relationship between autophagy and cellular senescence. Cellular senescence has been described in the conventional outflow pathway from glaucomatous donors, and in glaucomatous TM primary cultures [158]. Similarly, cells exposed to the hyperoxic environment showed elevated levels of the senescence marker, senescence-associated-β-galactosidase (SA-GLB1/SAβ-gal) [156]. SA-β-gal is an abnormal activity of the lysosomal β-galactosidase that is detected at a more basic pH (pH ~6.0) in senescence cells, which is thought to reflect the increase lysosomal mass in ageing cells [159,160]. The occurrence of SA-GLB1 in stressed cells was partially blocked in the presence of the autophagy inhibitor, 3-methyladenine (3-MA) [155]. Moreover, trehalose treatment, but not rapamaycin, triggered SA-GLB1. Together, data suggested that SA-GLB1 activity occurs through MTOR-independent autophagy, and results from reduced autophagic flux within autolysosomes.

### 7.5 Autophagy in the outflow pathway of ocular hypertensive and glaucomatous eyes

Autophagy in human glaucoma has just been investigated in vitro so far. Primary cultures of TM cells isolated from glaucomatous eyes displayed lower protein levels of LC3-II and SQSTM1 [161]. Autophagy flux was not evaluated in this study to determine whether that data was a reflect of increased flux or inhibited autophagy; however, glaucomatous cells showed the constitutive activation of MTOR signaling, based on the increase in phosphorylated ribosomal protein S6 kinase beta-1 (RPS6KB), a downstream substrate of MTOR signaling. Moreover, glaucomatous TM cells failed to activate autophagy when subjected to a hyperoxic environment, and a decreased in lysosomal proteolysis was observed. These studies support the hypothesis of a biphasic autophagy response in the outflow pathway, similar to what has been proposed in other diseases (i.e Alzheimer disease, cancer) [162]. Activation of autophagy might occur early in the disease as protective mechanism in response to stressors (i.e oxidative damage, mechanical stress), but compromised later on, leading to a decline in autophagic function. Evidence of autophagy lysosomal dysfunction in human glaucoma *in situ* are indirectly supported by the higher levels of SA-GLB1 [158,161]. Also, ocular hypertension and glaucoma are common ocular clinical findings in patients affected with a subset of lysosomal storage disorders [163].

### 7.6 Autophagy in the outflow pathway of ocular hypertensive mouse models

Autophagy has been investigated in different mouse models of ocular hypertension. Defective autophagy flux has been recently reported in the myocilin (MYOC) ocular hypertensive mouse model [164]. These mice express a misfolded mutant form of human *MYOC* (*MYOC*^Y437H^), which causes early-onset glaucoma presumably by triggering ER-stress. The authors found evidence of autophagy activation in the outflow pathway cells of mice carrying the mutant form; however, autophagy flux was deemed to be impaired, based on the higher SQSTM1 levels observed in the transgenic mice. Genetic or pharmacological inhibition of autophagy exacerbated the intracellular accumulation of mutant myocilin and further elevated IOP levels. In contrast, stimulation of autophagy using tat-BECN1 peptide or torin promoted degradation of MYOC and reduced IOP. Impaired autophagy in the *MYOC*^Y437H^ mice was associated with chronic ER stress and induction of the transcriptional factor CHOP (C/EBP homologous protein). Deletion of CHOP restored autophagy function and enhanced the recognition and autophagic degradation of mutant myocilin.

Chronic ER-stress has been additionally implicated in the pathogenesis of glucocorticoids-induced OHT, which can occur in patients undergoing prolonged glucocorticoid treatment. Evaluation of autophagy in a murine model of glucocorticoids-induced OHT showed the upregulated expression of LC3 and BECN1, and downregulation of SQSTM1 in outflow pathway cells, suggesting autophagy activation [165]. Along the same lines, studies conducted by another research group found that intraperitoneal injection of rapamycin further activated autophagy and prevented IOP elevation in mice [166]. However, these results contrast those recently reported by Sbardella et al. [167] showing enhanced turnover of the ULK-1 autophagy initiation complex and subsequent inhibition of autophagy in dexamethasome-treated TM cells.

Hirt et al. investigated the autophagy lysosomal system in the iridocorneal region of DBA/2J mice [168]. At 5-6 months of age, DBA/2J develop ocular abnormalities similar to those observed in human pigmentary glaucoma, including iris atrophy, pigment dispersion and elevated IOP. IF and WB analyses showed increased SQSTM1 and LC3-II, but lower LAMP1 levels in the outflow pathway cells of DBA/2J mice. The research group generated DBA/2J::GFP-LC3, which ubiquitously express GFP-LC3. Interestingly these transgenic mice develop higher IOP elevation compared to their DBA/2J littermate controls. Electron microscopy also showed the increased content of pigment-loaded vesicles and autophagic figures in TM cells. Authors postulated that inefficient autophagic clearance due to the uncoupling between autophagy induction and lysosomal could be one of the underlying pathogenic mechanisms in the DBA/2J mice. This needs to be confirmed.

## 8. Autophagy in the neural retina and its role in glaucoma

### 8.1 Brief introduction to neural retina

Retinal ganglion cells (RGCs) are the primary output neurons in the retina that process the visual information and transmit it to the visual processing centers in the brain. RGCs are located in the inner surface of the retina, the ganglion cell layer, and extend their axons gathering at the optic disk, where they become myelinated forming the optic nerve. Progressive loss of RGCs and axonal degeneration are the hallmark of glaucoma, a complex, multifactorial disease, which is the leading cause of irreversible blindness worldwide. Although, aging and elevated IOP have been identified as main risk factors for developing glaucoma, the pathogenesis of this neurodegenerative disease remains unclear and is likely caused by the combined effects of several molecular pathways. Therefore, identifying the mechanisms that are involved in the death of RGCs or those that support their survival is key for developing new therapeutic strategies.

### 8.2 Autophagy in RGC death in glaucoma: friend or foe?

Given the intrinsic limitations that hinder the study of glaucoma in humans, several animal models have been developed to investigate the molecular mechanisms underlying disease progression. Each model has its specific advantages and disadvantages related to the animal species used and the type and magnitude of the insult, and none completely replicate the human disease. The inbred DBA/2J mouse displays elevated IOP and spontaneously develops an optic neuropathy that is very similar to human glaucoma and is characterized by RGC degeneration [169]. In other models of glaucoma, IOP is elevated by injecting particles or hypertonic saline solutions, or by inducing episcleral vein coagulation [170,171]. In models of normal tension glaucoma, RGC death is induced by injection of toxins such as NMDA [170] or by direct optic nerve damage (e.g. by nerve crush or axotomy) [172]. Combination of some of these techniques with autophagy reporter animals allow for in vivo study of autophagy modulation in experimental glaucoma. Modulation of autophagy is a recurrent and functional response of RGCs to IOPdependent or independent glaucoma-related insults [173]. However, in RGCs, autophagy has been found to take part to either neuroprotection as well as neuronal death depending on the experimental setting (i.e. animal model, timing of the experiments, drugs/doses used to modulate the pathway).

#### 8.2.1. RGC autophagy under chronic ocular hypertension

In the optic nerve of DBA/2J mice, a spontaneous ocular hypertensive model of glaucoma, Coughlin and colleagues reported an increased number of autophagosomes, with upregulation of LC3-II/LC3-I ratio [174]. In the retina of these mice, an overall decrease of the autophagic flux, with downregulation of LC3II, SQSTM1 and LAMP1 was shown [168]. Interestingly, DBA/2J:GFP-LC3 mice developed an early and significant higher IOP compared to DBA/2J and the expression of the GFP-LC3 transgene caused greater RGC degeneration with axon swelling and massive presence of autophagic vacuoles [168]. A similar observation was reported by Nettesheim and colleagues in aged GFP-LC3 mice subjected to unilateral elevation of IOP by injection of hypertonic saline into the limbal vein; the transgene was associated with a greater magnitude of RGC death and axonal degeneration compared to wild type mice [154]. In a chronic model of ocular hypertension induced by episcleral veins cauterization the number of autophagosomes detected in the soma and dendrites of RGCs was enhanced and the LC3-II/LC3-I ratio and BECN1 expression were upregulated [175,176]. Treatment with the autophagy inhibitor 3-MA prevented cell loss in the ganglion cell layer suggesting that autophagy promoted RGC degeneration [175,176]. However, in another study, induction of autophagy by rapamycin was associated with reduced RGC loss following cauterization of episcleral veins [177]. Similarly, intravitreal rapamycin treatment enhanced RGC survival in rats subjected to circumlimbal suturing [178]. Upregulation of LC3-II and accumulation of autophagic vacuoles were detected in the retina of primates and rats with chronic ocular hypertension established by laser photocoagulation [179,180]. In this model inhibition of autophagy by 3-MA amplified RGC axonal degeneration while induction of autophagy by rapamycin was neuroprotective [180]. A neuroprotective role of mitophagy was also reported in the chronic ocular hypertension model established by injection of micro-magnetic beads into the anterior chamber of the eye [181]. Using the same model, Zhang et al. found that the effect of autophagy modulation on RGC survival was time-dependent. Inhibition of autophagy early after the establishment of ocular hypertension reduced RGC loss, while a delayed treatment with 3-MA or chloroquine (CQ) was associated with increased RGC death [182].

#### 8.2.2 RGC autophagy under acute ocular hypertension

Alteration of the autophagic flux has been shown in the retina of rodents subjected to acute OHT. However, the timing of ATG-related protein changes following the insult varied significantly among the studies. Accumulation of LC3-II and autophagosomal structures were reported in the soma of RGCs between 6 and 24 hours after the transient elevation of IOP [183-186]. More recently, Russo and coworkers showed a time-dependent modulation of autophagy characterized by a peak of autophagy activation in the first hours after the insult followed by a decline of autophagic turnover with accumulation of SQSTM1-positive bodies and a autophagic compartments in the soma of RGCs [187]. In the latter study, the decrease of RGC survival observed in mice with a genetic impairment of basal autophagy due to the heterozygous ablation of *Ambra1* (autophagy and beclin 1 regulator 1) (*ambra1^+/gt^*) supported the neuroprotective role of the endogenous autophagy response following the insult. According to a prosurvival role of autophagy in mice subjected to acute ocular hypertension, induction of autophagy by either sub chronic systemic treatment with rapamycin or 48h fasting prevented RGC loss [187]. Conversely, in the same model established in rat, acute intravitreal administration of rapamycin did not provide neuroprotection [184] and treatment with the autophagy inhibitor 3-MA partially prevented neuronal loss in the ganglion cell layer [183].

#### 8.2.3 RGC autophagy after optic nerve crush/transection

Upregulation of BECN1 and LC3-II were described in the retina and isolated RGCs following optic nerve (ON) transection in mice [188]. More recently, opposite results were reported by Oku and colleagues (2019) showing a decrease of LC3-II/LC3-I ratio and increase of SQSTM1 implying an impairment of autophagy [189]. Although the attenuation of axonal degeneration observed in rat subjected to ON crush following intravitreal injection of 3-MA would suggest that, under some experimental conditions, autophagy may be part of the degenerative process triggered by ON lesion [190], most of the available data leads to opposite conclusions. The neuroprotective role of autophagy in axotomized retina was demonstrated by Rodriguez-Muela and colleagues showing that RGC-specific deletion of *Atg5* or *Atg4B* knock down reduced RGC survival while rapamycin treatment partially prevented RGC loss [191]. Interestingly, following the lesion of ON in rat, induction of autophagy by *Sqstm1* silencing (which prevents SQSTM1 accumulation facilitating autophagy) offered better preservation of visual function and greater prevention of RGC apoptosis compared to rapamycin treatment, due to the selective deactivation of MTORC1 without inhibition of MTORC2-mediated positive effects [192]. More recently, an agedependent increase of RGC vulnerability following ON crush was observed in mice with deficient autophagy [193]. Indeed, the heterozygous ablation of *Ambra1*, a positive regulator of autophagy initiation [194], was associated with a decline of RGC survival in middle age mice (12-14 months) compared to age-matched wild type littermate [193].

### 8.3 Mitophagy, Optineurin and normal tension glaucoma

Mutations in *OPTN* (optineurin) and *TBK1* (TANK-binding kinase 1) genes, have been associated to normal tension glaucoma, which give rise to glaucomatous neurodegeneration in the absence of increased IOP [195-197]. OPTN is an autophagy receptor that harbours an LIR, which connects the ubiquitinated autophagy substrates with LC3 in autophagosomal membranes [198]. Cargoes that are degraded via OPTN include mitochondria, intracellular pathogens, and protein aggregates. Phosphorylation of OPTN by TBK1 enhances its binding to ubiquitinated chains and promotes selective degradation of mitochondria [199]. Interestingly OPTN also participates in autophagosome formation, recruiting the ULK1 complex to initiate phagophore formation, and also recruiting ATG9 [200-202]. Mice expressing the E50K mutation in *Optn* display a glaucoma phenotype characterized by RGC loss leading to thinning of the nerve fiber layer and the entire retina [203]. This mutation alters the interaction of OPTN with Rab8 and leads to reduced autophagy and increased oxidative stress [204,205]. Other studies have investigated how these mutations impact PINK1 (PTEN-induced kinase 1)-Parkin dependent mitophagy [201,206]. One such study demonstrated that expression of mutated E50K optineurin induces mitochondrial fission and mitophagy in axons of the RGCs of aged E50K−tg mice *in vivo* [207]. However, *in vitro* studies in cells expressing mito-Keima, a reporter of mitophagy flux, reported no mitophagy defects in cells expressing several glaucoma-associated optineurin mutations [208]. Thus, further studies are required to determine the impact of optineurin mutations for mitophagy in human glaucoma as well as in other diseases [209].

### 8.4 Autophagy in axonal degeneration in traumatic injury and experimental glaucoma models

Just a limited number of studies have investigated the role of autophagy in ON degeneration in glaucoma [190,210-212]. Although these studies suggest that autophagy might have a higher role in axonal degeneration than soma death, its function still remains unclear. Studies conducted using the traumatic injury model indicate an increase in the protein levels of LC3 and SQSTM1, as well as in the number of intra-axonal autophagic features soon after injury [190,210]. Both, pharmacological inhibition of calcium influx or autophagy attenuated axon degeneration, suggesting a role of calcium influx in triggering autophagy after optic nerve crush [190]. In contrast, the study by Koch et al. [210], using the same model, pointed towards defective autophagy flux, rather than activation of autophagy, and showed that ROCK2 (Rho associated coiled-coil containing protein kinase 2) downregulation promoted axonal regeneration by increasing autophagic flux. Defective autophagic flux has also been implicated in axonal degeneration in ocular hypertensive rats [180] and in the DBA/2J mouse glaucoma model [174,213]. Similar to traumatic lesion, chronic elevation in IOP led to an intraaxonal increased in autophagic figures and autophagosomes markers. Single treatment with rapamycin, which exerted a neuroprotective effect, further elevated LC3II levels, but decreased SQSTM1, which it was interpreted, but not confirmed, as enhanced autophagic flux [180]. The distribution of autophagic vesicles and mitochondria in the proximal and distal ON axons in the DBA/2J mice was investigated by serial block face scanning electron microscopy in [174,180]. The authors reported potential defective mitophagy in the glaucomatous optic nerve, caused by failure of anterograde transport of autophagic vesicles, contributing to distal axonopathy.

### 8.5 Modulation of autophagy as neuroprotective in glaucoma

The relevance of autophagy as an endogenous neuroprotective mechanism activated by RGCs is a finding overall consistent in several animal models of glaucoma. However, as seen earlier, autophagy induction under some experimental setting exacerbated RGC loss. These contrasting results might be reconciled by the hypothesis that the role of this pathway, over the course of RGC degeneration, is time-and insultdependent. This would suggest that in clinical glaucoma the timing of autophagy modulation might rely on the state of the disease and it would be a crucial factor for the efficacy of a potential therapy. Furthermore, most of the experimental studies focused on the induction of autophagy by the mTOR inhibitor rapamycin which, owing to its mechanism of action, may exert several additional effects making more difficult a clear interpretation of the data. Several molecules, developed or repurposed, have been identified as autophagy enhancers; some of these drugs act by activating AMPK (e.g. metformin, simvastastin, trehalose,), lowering inositol and inositol 1,4,5-trisphosphate (IP3) levels (e.g. lithium, carbamazepine), activating sirtuins (e.g. resveratrol) or modulating TFEB (e.g. curcumin-C1, cinnammic acid, luteolin, spermidine) [214,215]. Testing selective and mTOR-independent autophagy modulators in the different animal models of glaucoma will help gaining relevant information which are needed to further explore the clinical feasibility of targeting autophagy for retinal neuroprotection.

## 9. Autophagy in the Retinal Pigment Epithelium and its role in Age-Related Macular degeneration

### 9.1 Structure and Function of the Retinal Pigmented Epithelium

A polarized pigmented epithelial cell monolayer, the highly specialized retinal pigment epithelium (RPE) forms a critical barrier between the neural retina and choriocapillaris. The healthy RPE performs essential functions necessary for the maintenance and survival of the overlying photoreceptors [216]. Given that the RPE is comprised of terminally differentiated cells, autophagy processes are essential for cellular quality control, as these cells are unable to decrease their load of damaged organelles, accumulated lipids or protein aggregates through cell division [217-219]. Autophagy pathways (canonical, non-canonical or selective) must work in coordination to maintain RPE and retinal homeostasis by maintaining (1) visual pigment balance, (2) efficient phagocytosis and lysosome mediated degradation, (3) metabolic homeostasis, and (4) organelle function through mitophagy and autophagy of peroxisomes (pexophagy). Moreover, RPE autophagy is an adaptive, stress response that when dysregulated or altered during aging contributes to pathophysiological conditions associated with age-related macular degeneration (AMD).

This section will focus on the most recent progress on the role of autophagy in RPE health and dysregulation in autophagy as contributing to AMD. We discuss potential therapeutics that may be developed based on these scientific advances.

### 9.2 Autophagy in RPE metabolic homeostasis

The necessity for highly regulated autophagic processes in the RPE becomes clear when one considers the intimate functional relationship between the RPE and photoreceptors in maintaining visual function [216]. In the central mouse retina, through a daily synchronized burst of phagocytic activity, each RPE cell can ingest the shed distal tips of over 200 photoreceptor cells [220]. The protein- and lipid-rich ingested outer segments (OS) provide a bolus of fatty acids that through mitochondrial fatty acid βoxidation (FAO) generates energy for the RPE [221,222]. RPE FAO also generates substrates for ketogenesis; ketone bodies are released apically to provide energy to photoreceptors [222]. The reliance of the RPE on oxidative metabolism also spares glucose for use by the neural retina [223]. Glucose is transported across the RPE from the choriocapillaris to the outer retina where it is metabolized by aerobic glycolysis to generate lactate. RPE takes up lactate from the subretinal space and uses the lactate to fuel oxidative phosphorylation. In addition to oxidizing lactate, the RPE, on a daily basis oxidizes OS derived fatty acids. This high flux through oxidative phosphorylation puts a tremendous burden on mitochondria and peroxisomes (necessary to degrade the long-chain fatty acids in ingested OS). If the OS is unable to adequately degrade and metabolize OS fatty acids due to defective phagosome maturation/degradation and/or lysosome dysregulation, the RPE is predicted to rely on glucose metabolism, thus depriving the outer retina of its much-needed energy source. Indeed, a recent study demonstrates that a slight reduction in autophagy, as it is observed in the *Ambra1*^+/gt^ mice, in both the retina and the RPE causes early alteration in the RPE that result in a pro-aging phenotype with reduced vision and important metabolic alterations in the retina and RPE [224]. *In vitro* studies document that high glucose results in an increase in autophagosomes and altered expression of LC3 and SQSTM1 [225], suggesting that autophagy plays a protective role under glucose stress [225,226]. In *vitro* studies suggests that the effect of glucose on RPE autophagy is dose dependent; high-glucose (50mM) inhibits cell mitophagy while low-glucose (15mM) induces cell mitophagy [227]. Stress conditions, such as high glucose also induce inflammasome signaling in RPE cells upon defunct autophagy [226]. Whether glucose metabolism regulates RPE-associated autophagy *in vivo* needs to be investigated.

**Figure 6.**
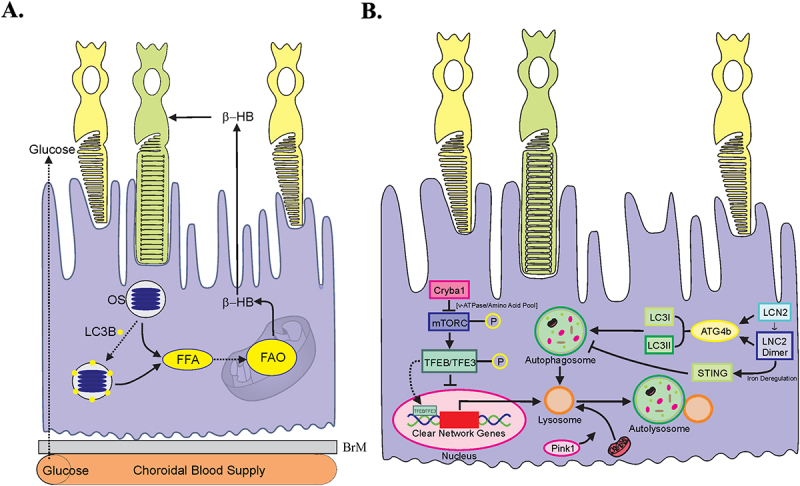
**Schematic representation of autophagy in RPE metabolic health and inflammation. (A)** Schematic representation depicting the interrelationship between LC3B associated phagocytosis and RPE- fatty acid oxidation. Oxidation of OS derived fatty acids provides B-Hydroxybutyrate (B-HB) and spares glucose for use by the photoreceptor rod cells as depicted. **(B)** Autophagy deregulation in *Cryba1* cKO RPE cells. In the RPE cells, the lysosomal luminal proteinβA3/A1-crystallin (encoded by *Cryba1* gene) binds to vATPase and PAT4. Loss of βA3/A1-crystallin specifically in the RPE (*Cryba1* cKO mice) leads to increased lysosomal pH, elevated levels of cytosolic amino acids, activation of mTORC1 signaling, decline in TFEB nuclear translocation and CLEAR network gene expression, along with a decrease in basal autophagy, including mitophagy. Further, in the RPE cells from *Cryba1* cKO mice and human AMD donors, there is an elevated level of the LCN2 homodimer, a pro-inflammatory adipokine, which unlike its monomeric counterpart, has a higher half-life in the body and is unable to chelate iron. On the other hand, the LCN2 monomer is required for autophagy regulation, as it can form a complex with ATG4BLC3, thereby regulating ATG4B activity and LC3-II lipidation in the RPE cells. However, in the diseased state, the upregulation of the homodimer variant triggers accumulation of iron and subsequent activation of the cGAS/STING-mediated inflammasome pathway. The increased level of the homodimer also blocks the complex formation with ATG4B-LC3, resulting in altered LC3 lipidation and compromised autophagosome processing.

The reoccurring daily phagocytosis and degradation of OSs over the lifetime of the post-mitotic RPE presents a unique set of autophagy process challenges for these cells. The RPE relies on canonical autophagy (CA) to protect against light and oxidative stress, and on a hybrid autophagy-phagocytosis degradative pathway, termed LC3-associated phagocytosis (LAP, more extensively discussed in Section 12) for degradation of OS [228,229]. Mitophagy and pexophagy provide quality control to ensure healthy mitochondria and peroxisomes. These quality control processes are not trivial given that in the human retina, Volland, et al. [220] estimate that each RPE ingests ~186 million molecules of rhodopsin daily, this corresponds to ~0.08-0.15 pmoles of fatty acid for FAO per RPE, similar to metabolic load in hepatocytes in western diets. A similar phagocytic load and hence fatty acid oxidative capacity is predicted in the central region of the mouse retina [220]. The interrelationship between LAP and metabolic synergy between the RPE and neural retina is illustrated in Figure 6A.

In contrast to CA, LAP is a ULK, nutrient- independent process, requiring Atg5 and melanoregulin to aid in the lipidation of ingested phagosomes [229]. Three genes encode the highly homologous, LC3 protein family members, MAP1LC3A (LC3A), MAP1LC3B (LC3B) and MAP1LC3C (LC3C) [230]. LC3B predominates in both fetal and adult human RPE, while in mouse RPE and neural retina, LC3A and LC3B were expressed at approximately equivalent levels [230]. Whether these LC3 isoforms are functionally redundant or play a specialized role(s) in maintaining RPE homeostasis is not known. LC3B isoform is associated with LC3B associated phagocytosis (LAP). Deletion of LC3B in the *lc3b^−/−^* mouse retains CA, but inhibits LAP. Mouse models of defective LAP exhibit disrupted lipid homeostasis: decreased levels of lipid pro-survival factors, increases in neutral lipid deposits and in the pro-inflammatory sterol, 7ketocholesterol (7KCh) [229,231], an oxysterol present in high levels in drusen. When the ability of RPE to remove “waste” is compromised, as in the absence of LAP, the generation of 4-HNE, 7KCh and other lipid peroxidation by-products [229,231] are predicted to induce a low grade inflammatory response, RPE damage and death [232-234].

Clues to how these various autophagy processes are regulated come from our understanding of the circadian and light-mediated regulation of autophagy associated proteins, which will be more extensively discussed later in Section 11. In brief, the daily burst of OS phagocytosis is accompanied by an increase in LC3, as well as a bimodal conversion of LC3-I to LC3-II, which fluctuate over the 12 h light/dark cycle [228,229,235]. ATG5, ATG7 and ATG12 expression levels are also cyclic [228,229,235]. On a molecular level, critical player coordinating CA and LAP are RUN (RPIP8/UNC-14/NESCA) and rubicon (RUBCN). RUBCN activation commits a cell to LAP, while concomitantly inhibiting CA [236]. Interestingly, a recent study suggests that miR-211 modulates autophagy at lights on by targeting ezrin and stimulating lysosomal biogenesis [237]. The relationship between RUBCN and miR-211 as a means by which to prepare the RPE for LAP mediated OS degradation/metabolism is an intriguing possibility.

### 9.3 Autophagy in inflammation

On a molecular level, inhibition of autophagy activates inflammasomes that triggers oxidative stress and can lead to RPE cell death [238]. Indeed, many autophagy deficient animal models display increases gliosis that is also exacerbated with age [224]. Inflammasomes are multiprotein signaling complexes that serve as platforms for the activation of caspase-1 and the release of mature IL-1β and IL-18 [239]. In addition to cytokine activation, caspase-1 also participates in cell death called pyroptosis [240]. Several pathogen-recognition receptors, such as NOD-like receptors [NLRs; nucleotide-binding domain, leucinerich repeat (LLR)-containing proteins], C-type lectin receptors, retinoic acid-inducible gene-I-like receptors, and cytosolic DNA sensors, can form inflammasomes. Currently, the most extensively studied and the best characterized inflammasome receptor is NALP3 (NLR family pyrin domain containing 3, also called cryopyrin, caterpillar-like receptor 1.1, CIAS1, or PYPAF1) [241]. The presence of NLRP3 (NOD-, LRR- and pyrin domain-containing protein 3) inflammasome in RPE cells was shown for the first time in 2012 [242,243]. Recently, mitochondrial damage is suggested to play a role in AIM2 inflammasome in human RPE cells [244]. Extensive evidence suggests that autophagy and inflammasome regulate each other. Autophagy-related proteins are associated with inflammasome activation, and lysosomal rupture with the release of cathepsin B into the cytosol; one of the main mechanisms of NLRP3 inflammasome activation [245-247]. By degrading aged mitochondria in a process called mitophagy, autophagy alleviates oxidative stress and the release of mitochondrial DNA (mtDNA) to the cytosol both of which serve as activators for inflammasome signaling [246,248]. In addition to preventing inflammasome formation, autophagy is needed to remove inflammasome components as well as pro-forms of IL-1β and IL-18 when unnecessary or excessively expressed [249-253]. Conversely, RPE cell swelling caused by the over-expression of pro-IL1β and pro-IL-18 can be abrogated by autophagy activation [254]. In experimental models, several stress conditions, such as proteasome inhibitors induce inflammasome signaling in RPE cells upon defunct autophagy [255]. Dysfunctional autophagy in RPE cells has also been shown to induce inflammasome activation in infiltrating macrophages [233].

### 9.4 Autophagy and Age-related macular degeneration

The RPE has been identified to be among the first cells affected in human dry AMD [256]. A growing body of evidence suggests that reduced autophagic activity in the RPE is linked to the development and progression of AMD. However, even though activation of autophagy is beneficial for the damaged RPE, excessive autophagy can also lead to retinal cell death, suggesting that a fine regulation of this process is required [257]. A major contributor to the development of AMD is oxidative stress, which progresses with age. Indeed, RPE is continuously exposed to light, high concentrations of polyunsaturated fatty acids (substrates for generation of ROS) and photosensitizers [258]. With age, increased deposition of lipids and proteins in the RPE and at the Bruch’s membrane occurs; and accumulation of lipofuscin within the RPE promotes the formation of additional free radicals thus exacerbating oxidative stress. *In vitro* studies have shown that while acute oxidative stress promotes autophagy, chronic oxidative stress leads to a reduction of autophagic activity [259]. This was also observed in RPE cells from AMD donors [260]. Moreover, increased levels of lipofuscin, together with increased ROS and metabolic alterations were observed in *Ambra1^+/gt^* mice compared to wt littermates. Importantly, those animals display higher sensitivity to the sodium iodate model of experimental AMD, showing increased photoreceptor apoptosis and decreased retinal thickness [224]. Lastly, inhibition of autophagy in ARPE-19 cells facilitated accumulation of lipofuscin and generation of ROS, while promoting autophagy has the opposite effect, suggesting that autophagy is beneficial for fighting chronic oxidative stress observed in AMD [261]. The molecular mechanisms of autophagy-mediated protection from oxidative stress are reviewed in detail in [257]. It was recently proposed that telomerase, an enzyme that elongates telomeric DNA, may serve as an additional link between autophagy, oxidative damage response and cell senescence [reviewed in [262]]. Human hTERT (telomerase reverse transcriptase), the catalytic subunit of telomerase, was shown to directly interact with autophagy regulators, including mTORC1. Previously, βA3/A1-crystallin, a lysosomal lumenal protein in the RPE, has been shown to be necessary for the crosstalk between amino acids, V-ATPase and MTORC1, thereby regulating autophagy in the RPE [263]. Furthermore, telomerase prevents senescence in proliferating cells and extends telomeres that are particularly sensitive to the oxidative stress due to their high guanine content [262]. Thus, targeting telomerase might be a promising therapeutic approach aimed at fighting the devastating consequences of chronic oxidative stress, such as the decrease in autophagy, shortening of telomeres and induction of cell senescence.

Autophagy deficiency caused by deletion of *Atg5* or *Atg7* in *atg5ΔRPE* and *atg7ΔRP*E mice resulted in uneven RPE thickness, RPE hypertrophy/hypotrophy, pigmentary irregularities and choroidal neovascularization. The severity of the phenotype increased with age [264]. As expected, inefficient degradation of phagocytosed OS and disrupted visual cycle were also observed in autophagy-deficient RPE [230]. Decreased autophagy along with accumulation of lipofuscin in the RPE activated inflammatory response, which in turn contributed to the establishment of chronic inflammation and acceleration of cell senescence, resulting in the development of AMD [reviewed in [134]]. Recently, LCN2 (lipocalin-2), a known inflammatory adipokine, was shown to form a complex with both ATG4B and LC3B, thereby regulating autophagosome maturation. RPE cells that have increased LCN-2 exhibit decreased autophagy flux. This autophagy dysregulation leads to an abnormality in iron homeostasis, causing the elevation in the levels of redox-sensitive iron, resulting in inflammasome activation, oxidative stress, and ferroptosis in the RPE cells. Interestingly, a monoclonal antibody (Clone #6 mAb) directed against LCN-2 could rescue the autophagy/inflammasome/ferroptosis processes, along with retinal function, in a dry AMD-like mouse model [265]. These processes are summarized in Figure 6B.

The role of autophagy in AMD cannot be discussed without paying special attention to a specialized type of autophagy aimed at degradation of defective mitochondria – mitophagy. RPE and photoreceptors both contain higher than average numbers of mitochondria [257], which are both a source and target of chronic oxidative stress. Moreover, senescent cells that accumulate with age contribute to additional mitochondrial damage. PINK1 is the essential regulator of mitophagy, which is a selective form of autophagy aimed at the removal of the damaged mitochondria [266]. Dysfunctional mitochondria that fail to be cleared by mitophagy, a process that becomes less active with age, release factors contributing to inflammation in the retina and AMD pathophysiology [reviewed in [267]]. In response to the daily degradation of OSs, a healthy pool of functional mitochondria and peroxisome are also necessary to generate essential metabolites. Diminished mitophagy has been reporter in the mouse model of dry AMD NFE2L2/PGC1α double knockout (dKO) mouse [268]. Potentially, targeting mitophagy and *de novo* biogenesis of fresh mitochondria might be a promising therapeutic strategy for delaying or preventing AMD.

## 10. Autophagy in photoreceptors and retinal diseases

### 10.1 Photoreceptor structure and function: Why is autophagy relevant

Photoreceptors (PRs), specifically rods and cones, are specialized light-sensitive neurons that serve as the initial components of the visual system. They are found at the back of the retina adjacent to the RPE [269-271]. The human retina contains approximately 120 million rods and 6 million cones, the latter of which are highly concentrated in the fovea. Rods are primarily important for vision under dim light conditions, as they are extremely light sensitive. Cones, on the other hand, function in bright light and are responsible for high acuity and color vision. Both rods and cones are structurally compartmentalized and are composed of five main regions; the outer segment (OS) which is used in light capture and its conversion into electrical signals (phototransduction), the inner segment (IS) which contains metabolic cell machinery and organelles (including mitochondria, endoplasmic reticulum, Golgi complex, and lysosomes), the connecting cilium which connects the OS and IS and allows for protein transport between them, the nuclear region which contains the nucleus, and the synaptic region which releases synaptic vesicles that allow glutamate (neurotransmitter) transmission from the PRs to bipolar cells.

In addition to the complex anatomic compartmentalization of the PR cells, there is a complex interaction of the PRs with the underlying RPE. Photoreceptors are highly metabolically active and require significant amounts of glucose and oxygen, supplied by the choroid and choroidal vessels via the RPE [272]. This metabolic demand stems from several factors, such as the incredibly high amount of protein synthesis and flux (both of *de novo* synthesized proteins, as well as of existing proteins that can translocate from one compartment of the cell to the other), visual transduction activities, and membrane synthesis. In addition, there is the flow of material from the PRs to the RPE. The OS are perpetually renewed; old membranes are shed from the distal end and new membranes are added at the proximal end [273]. The shed photoreceptor membranes are ingested by the RPE via phagocytosis.

The complex structure and function of the outer retina and the high metabolic demand of the PR cells requires a complex set of molecular machinery acting in a tightly regulated manner. Perturbations of these processes can result in significant disease, resulting in the need for a robust set of protective mechanisms to help maintain cellular homeostasis, with autophagy playing a central role in preserving PR cell integrity. Here, we will highlight some of the major principles regarding the role of autophagy in maintaining the health, or contributing to the disease, of the PRs.

### 10.2 Autophagy in photoreceptor cells

Given the substantial metabolic demands of PR cells, coupled with their highly complex compartmentalization and biologic function, it is crucial that PRs have access to nutrients that support the needed molecular machinery. Factors preventing or blocking nutrients from reaching the PR cells or resulting in altered homeostasis could lead to autophagy activation. Examples of factors which could lead to altered homeostasis include accumulation of cellular debris or damaged organelles, as well as the accumulation of excess or aggregated proteins leading to proteotoxicity. Photoreceptors utilize autophagy to maintain cellular homeostasis in response to environmental stressors (e.g., light activation, oxidative stress) [235,274-276]. Perturbations along any of the PRs’ normal biological pathways can result in disease, and it is becoming increasingly clear that autophagy helps in the maintenance of the integrity of these pathways. A prototypical example of the role of autophagy in maintaining PR homeostasis is its activation in response to changes in light conditions. In PRs, activation of autophagy by light is thought to be caused, at least in part, by the translocation of transducin from the OS to the IS [235]. In the dark, transducin is primarily located in the OS in proximity to the visual pigment rhodopsin and translocates to the IS upon light onset in one aspect of PR desensitization. The opposite is true for arrestin. Yao and coworkers predicted that PRs deficient in transducin would exhibit decreased activation of autophagy after light onset due to the decrease in protein entering the IS. Conversely, they predicted that the dark peak of autophagy activation would be decreased in the absence of arrestin, as there would be less arrestin translocating into the IS from the OS. Analysis from two mice strains deficient in either transducin or arrestin (*Gnat1*^−/−^ and *Sag*^−/−^ strains, respectively) bore out these predictions, and demonstrated that the translocation of transducin and arrestin proteins into the IS contributes significantly to the light- and darkassociated peaks in autophagy activation, respectively. Thus, in a 24-hour period, activation of autophagy displayed a bimodal pattern consistent with light-induced shifts in proteins within the PR cells. Conversely, in a mouse strain with a conditional knockout of Atg5 in the rod photoreceptors, the degeneration induced was reduced if either transducin or arrestin were also deficient, further evidence of the important role of autophagy in reducing proteotoxicity [277]. This contrasts with autophagy activation in the RPE, which they showed was not related to light/dark shifts *per se*, but rather to the circadian rhythm of OS shedding and ingestion by the RPE, discussed in Section11.

### 10.3 Autophagy in photoreceptor cell death: Can modulation of autophagy affect outcome?

Autophagy plays a vital role in maintaining PR homeostasis and function [278,279, 280]. Thus, it is not surprising that autophagy functions in regulating the response of cells to disease. One such example is the condition known as retinal detachment (RD), which occurs when the neurosensory retina becomes separated from the underlying RPE and choroid. As mentioned above, PR cells receive their oxygen and nutrition primarily from the RPE/choroid complex. Thus, during RD, the PRs are separated from their source of nutrition and autophagy is activated [281-283]. Studies have shown that in experimental RD, autophagy activation functions to improve PR survival. Inhibiting autophagy during RD results in increased PR cell death. One upstream activator of autophagy during RD is HIF (hypoxia-inducible factor), consistent with the induction of hypoxia by the detachment [284]. Activation of HIF-1α (but not HIF-2α) acts as an early response signal to activate autophagy and decrease PR apoptosis.

Clinical experience has shown that retinal detachments repaired within one week of symptom onset tend to have better visual outcomes than those repaired after one week. Chinskey and coworkers showed that there is a time-dependent increase in the activation of calpains within the retina after RD, with levels of these proteases peaking at approximately seven days post detachment [282]. Increased calpain activity contributed to the cleavage of ATG5, an upstream activator of autophagy, and thus decreased autophagy levels and increased PR cell death. These findings demonstrate how changes in autophagy levels within the PR affect cell death and help explain, at least in part, the clinical observations. Based on these findings, it was proposed that modulating autophagy following retinal detachment could improve survival [282,285]. Calpain inhibitors have been shown to be neuroprotective in multiple models of eye disease, including photoreceptor degeneration [282,286]. In an *in vitro* cell culture model, peak activation of Calpain 1 – the protease responsible for ATG5 cleavage and for inducing apoptosis in PRs – paralleled decreased autophagy and increased cell death [282]. In an experimental model of RD, calpain inhibitors were found to decrease the time-dependent activation of calpains, increase autophagy levels, and decrease activation of apoptosis and PR cell death, suggesting that calpains shift the PR cells from survival to death, at least in part, through their effect on autophagy. These observations support the importance of autophagy in promoting PR survival in low nutrient environments, as present during RD [281]. Based on this evidence, calpain inhibition has been proposed as a method for extending the period of autophagy, allowing for decreased apoptosis and prolonged cell survival. The data support a new approach towards neuroprotection and highlight modulation of autophagy as a vital avenue to explore for RD therapeutics [285].

Positive effects of autophagy have also been observed in some models of inherited retinal degeneration (IRD). Activation of autophagy via rapamycin treatment has been shown to improve retinal function and morphology ina mouse model of retinitis pigmentosa (RP) [287], a group of inherited vision disorders that cause progressive retinal degeneration. In contrast, conditional knockdown of *Uxt* (ubiquitously expressed prefoldin like chaperone), a positive regulator of mTOR activity, in the mouse retina resulted in severe retina degeneration resembling that in RP [288]. Similarly, CERKL (ceramide kinase like), one of the RP causative genes, was shown to negatively regulate autophagy by affecting the protein stability of SIRT1 (sirtuin 1), which deacetylates ATG5 and ATG7. Ablation of *cerkl* in zebrafish reduced the basal levels of autophagy in PR and RPE cells prior to PR degeneration [289]. In a very recent study, activation of chaperon-mediated autophagy was shown to ameliorate retinal degeneration in the *rd10* RP mouse model [290].

It is important to recognize, however, that activation of autophagy does not provide a beneficial effect across all retinal diseases. One example of this is highlighted in studies on the P23H form of autosomal dominant RP, a subcategory of IRD caused by mutations in the rhodopsin (*RHO*) gene that lead to protein misfolding [291]. The P23H variant of RHO results in protein misfolding that triggers ER stress, which is notably one of the activators of autophagy. The accumulation of misfolded protein and ER stress culminates in the destabilization of rod disk membranes leading to PR death [292]. Increasing the removal of misfolded protein, either by translocation from the ER to the proteasome or via degradation by autophagy, could serve as a basis for therapeutic intervention in this form of retinal degeneration. A 2018 study [293] sought to further investigate and understand the widely held view that increased autophagy was needed to clear misfolded rhodopsin and decrease PR cell death. When examining retinas from mice heterozygous for the RHO-P23H mutation, they observed that there was indeed greater autophagy flux in the PR cells. Based on the premise that further increasing autophagy might further decrease ER stress and enhance PR survival, they examined the effect of treatment with the autophagy activator CCI-779. The vehicle treated mice had no change and displayed the same pattern of PR degeneration. Surprisingly, CCI779 treated mice exhibited more rapid retinal degeneration and loss of outer nuclear layer thickness. In contrast, reducing autophagy flux – either therapeutically or by rod-specific deletion of the autophagyactivating gene *Atg5* – resulted in improved photoreceptor structure and function. The findings indicate that elevated autophagy may contribute to photoreceptor death in P23H mice, necessitating reexamination and retargeting therapeutic strategies that stimulate autophagy as a means to mediate retinal degeneration.

To explore the mechanism underpinning this paradoxical finding, the investigators postulated that the increase in autophagy was reducing activity of another protective pathway, proteasome. They demonstrated an increased ratio of autophagy flux to proteasome activity in the P23H retina and proposed its use as an index of altered photoreceptor cell homeostasis. Based on this, they hypothesized that interventions that normalize this ratio, by either reducing autophagy flux or increasing proteasome activity, will decrease PR death [294]. To test this, P23H mice were treated with either 4-phenylbutyric acid to enhance rhodopsin folding as a mechanism to decrease ER stress and autophagy activation, or with phosphodiesterase-4 inhibitor (rolipram) to increase proteasome activity. The P23H mice that received either treatment demonstrated decreased ER stress, decreased autophagy flux, increased proteasome activity, and decreased activation of cell death pathways. The rate of retinal degeneration decreased, and photoreceptor structure and function were improved as compared to control mice. The results suggest that limiting autophagy flux in photoreceptors of individuals with retinal degeneration caused by the P23H rhodopsin variant is an important avenue for further research, and further highlights the complex interaction of autophagy with other homeostatic regulatory systems in the cell such as the ubiquitin/proteasome pathway.

## 11. Diurnal/Circadian variation of autophagy in retinal physiology and disease

Autophagy occurs in rhythmic fashion in tissues and organs to optimize metabolism, nutrient balance and intracellular degradation in concert with feeding and light/dark cycles [295-297]. It has been proposed that autophagy rhythms in mammals can be distinguished by the status of targeted subcellular proteins: e.g. localization of cytosolic proteins and proteosomal degradation occurs via autophagy during the active phase of the day, while ER and mitochondrial removal occurs via autophagy during the resting phase. However, age-related changes in biological rhythms lead to reduced autophagic flux at a time when there is an increased need for removal of damaged proteins and organelles, thus contributing to neurodegeneration in the elderly [298].

As discussed earlier in this review, autophagy proteins are strongly expressed in the neural retina and RPE. Reme and colleagues were the first to report on diurnal rhythmicity in autophagy in the retina when they described increased autophagosome formation following outer disk shedding in rat photoreceptors [299]. Further study demonstrated that, under a light-dark cycle, autophagy in the rat retina peaks in the mid light phase and that this is under circadian control [300]. This is supported by Yao et al who observed that autophagy in photoreceptors and RPE cells of C57BL/6 mice maintained under a normal light/dark cycle conditions demonstrated a biphasic pattern of autophagy flux [235]. They concluded, by varying light cycle conditions, that photoreceptor autophagy is under circadian control, while RPE autophagy was independent of lighting conditions but appeared to correlate, at least in part, with the ingestion of outer segments. It should be noted that there is a rhythmic interplay between a “non-canonical” form of autophagy and the phagocytosis of photoreceptor outer segments within the RPE involving both LC3 and ATG5 [228,229]. It was subsequently reported that the expression of key proteins in the autophagy pathway of the mouse neural retina exhibit an intrinsic diurnal rhythm [301]. ATG9 and LC3 exhibited a significant biphasic 12/12 hour circadian cycle (light on at 6.00am) with peak at 8:15 AM and 8:15 PM and trough at 2:15 AM and 2:15 PM. By contrast, ATG7 and BECN1 expression showed a monophasic rhythm; peak at 8.15 am and trough at midnight [301]. Analysis of these autophagy proteins at their peak demonstrated that ATG9, LC3 and BECN1 were localized throughout the retina, predominantly within the RGC layer, inner (INL) and outer nuclear layer (ONL) and vascular endothelial cells while ATG7 was more strongly localized to the photoreceptors. BECN1 staining was detected throughout the retina, with highest intensity in the inner and outer plexiform layers and in the photoreceptors [301]. Furthermore, diurnal oscillations in autophagic activity in the murine retina depend, at least in part, on light-effected entrainment since the periodic oscillations were phase shifted and overall levels of autophagic proteins were reduced in dark-adapted mice retina when compared to a normal light/dark cycle [301]. Rhythmic cycling of autophagy has been reported in the retina for other species including Xenopus and porcine [302,303].

As discussed earlier in this review and elsewhere, dysregulated autophagy is strongly associated with retinal pathologies such as diabetic retinopathy [304,305] and AMD [257,306]. Dysregulation of circadian rhythm is strongly associated with diabetes and metabolism [307,308]. We observed a dramatic attenuation of diurnal rhythmicity (amplitudes) as well as overall levels of the autophagic proteins in the neural retina of mice with type 1 diabetes (T1D) and rats with type 2 diabetes (T2D) [301]. In the streptozotocin-induced T1D mice, this loss of amplitude and significant phase shift in the neural retina and vasculature was noted for ATG7, ATG9, LC3 and BECN1 expression in animals with both 2 and 9 months duration of diabetes. Analyses of autophagic proteins in the retina of T2D rats demonstrated not only suppressed levels but also revealed impairment in diurnal rhythmicity of autophagy proteins [301]. Expression of ATG9 and LC3 were severely suppressed with insignificant biphasic oscillatory pattern and ATG7 and BECN1 were phase-shifted by approximately for 4-6 hours. While there have been numerous studies linking circadian rhythm changes with AMD [309,310], to date, there have been no definitive studies showing that circadian dysregulation of autophagy is directly associated with the onset or progression of AMD. Similarly, autophagy dysregulation is associated with other ocular conditions such as corneal repair and glaucoma as discussed earlier in this review and these same pathologies are linked to circadian/diurnal disturbances [311-314]. In conclusion, further studies are required to determine the potential impact of dysregulation of autophagic rhythms on the onset and progression of these pathologies.

Given that autophagy levels in the normal PR fluctuate as a time of day and are triggered by background lighting conditions, it is important to take this into account when performing measurements of autophagy activity in the retina. Recording autophagy levels at multiple time points helps account for the dynamic nature of its activity and ensures that any changes detected are not due to a shift in the timing of the peak, but instead represent an actual change in the level of the peak resulting from the experimental conditions being tested.

## 12. LC3-associated phagocytosis and the visual cycle

In phagocytic cells a process termed LAP (LC3-associated phagocytosis) utilizes components of the autophagy machinery for optimal degradation of phagocytosed material. During LAP, lipidated LC3-II is recruited to the phagosome membrane to promote degradation by the lysosome. LAP and autophagy utilize the autophagy proteins ATG5, ATG7, ATG16L, ULK1, ATG13, FIP200 (RB1CC1), VPS34, and BECN1; while LAP is independent of the autophagy preinitiation complex consisting of ULK1/ATG13/FIP200 [315]. LAP is now recognized as important to the normal physiology and homoeostasis in many systems. Multicellular organisms utilize LAP to remove invading pathogens, degrade cell debris, and eliminate dead cells [315]. Although studied predominantly in murine and human cells, LAP has also been described in Zebrafish [316]. Defects in macrophage LAP can lead to enhanced proinflammatory cytokine production and autoimmunity [317]. Some bacteria actively inhibit LAP to promote their survival [318]; while LAP in tumor associated macrophages is suppressive to anti-cancer immunity [319]. Macrophages [320,321], plasmacytoid dendritic cells [322], and mammary epithelial cells [323] utilize LAP, as do the phagocytic RPE cells [228] for degradation of photoreceptor outer segments (POS) and the recovery of retinoids for the visual cycle.

The shedding and phagocytic clearance of POS is a daily process that helps to maintain healthy photoreceptors. Phagocytosis of the POS tips by the RPE, which are shed in the early morning, engages the LAP pathway that follows that same course as in other cell types. The engulfed POS enter the single membrane phagosome, the ATG12-ATG5-ATG16L complex is recruited, the lipidation of the cytosolic form of LC3 (LC3-I-LC3-II) occurs and is recruitment to the phagosome. Only then does the lysosome fuse to the phagosome forming the phagolysosome leading to degradation of the ingested POS cargo [228]. While LAP and autophagy are distinguished by the utilization of the autophagy preinitiation complex, there are also other features that separate these pathways. Canonical autophagy is characterized by a double-membrane autophagosome, whereas LAP is characterized by a single-membrane phagosome. In addition, MREG (melanoregulin) and RUBCN are specific for LAP and not required for canonical autophagy. MREG is a cargo-sorting protein that promotes the maturation of POS-containing phagosomes, outer segment degradation and nutrient recycling [229]. Loss of MREG leads to phagosome accumulation in the RPE. RUBCN also associates with the POS containing phagosome and is also required for optimal POS degradation. RUBCN expression in the RPE coincides with maximal disk shedding with its expression highest in the morning. Its loss also delays in POS degradation [324].

LAP is critical to RPE homeostasis as it may be required for the maintenance of normal lipid homeostasis [231]. LAP, and its associated mediators, also regulate the balance between autophagic and phagocytic degradation. RUBCN is a known inhibitor of autophagy and during POS phagocytosis its increased levels inhibit autophagy. Autophagy inhibition induced by POS phagocytosis also requires the participation of the EGFR (epidermal growth factor receptor). During phagocytosis engagement of the EGFR results in MTOR (mechanistic target of rapamycin [serine/threonine kinase]) stimulation, the accumulation of SQSTM1, and the phosphorylation of BECN1 on an inhibitory residue. These observations suggest the RPE regulate lysosomal pathways during the critical period of POS phagocytosis by suppressing canonical autophagy to conserve lysosomal resources which can be limiting [324].

**Figure 7.**
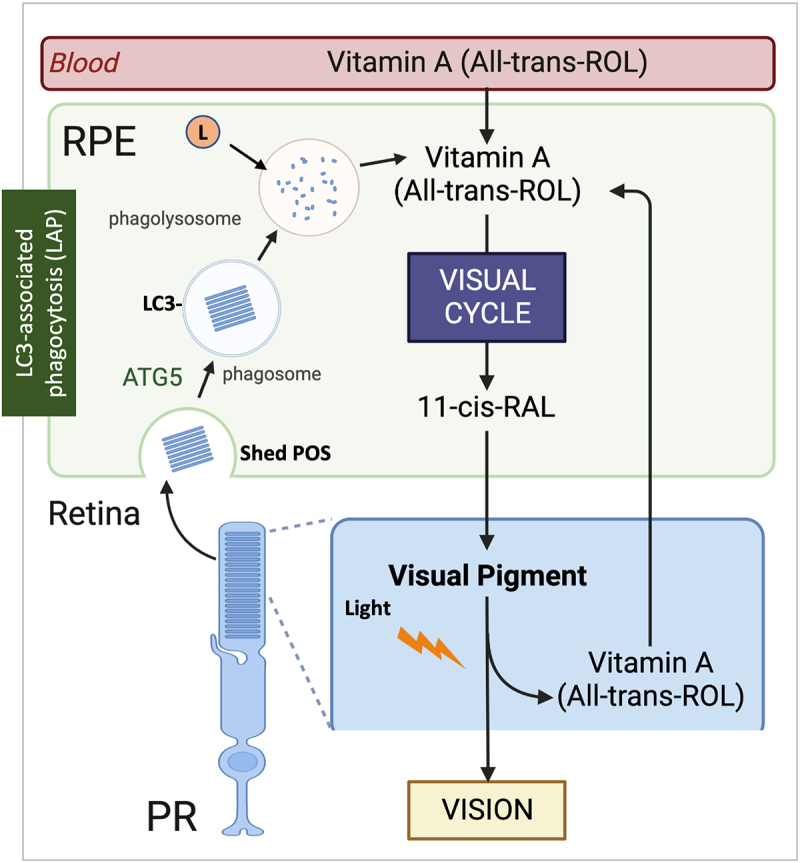
**LAP supports the RPE visual cycle**. The RPE performs two vital functions that sustain vision, phagocytosis of POS and the visual cycle. Phagocytosis and degradation of POS are supported by a noncanonical form of autophagy termed LAP. In this process, engulfed POS enter the phagosome, followed by the ATG5-dependent recruitment of the lipidated form of LC3. Only then does the lysosome fuse with the phagosome forming the phagolysosome leading to degradation of the POS cargo. The second critical process is the classic retinoid visual cycle that converts vitamin A (all-trans ROL) into the visual chromophore 11-cis-RAL. The visual cycle begins following the absorption of light in the photoreceptors by visual pigments containing the chromophore 11-cis RAL. This generates all-trans ROL (vitamin A) which is transported to the RPE for conversion back to 11-cis RAL by the visual cycle to recharge the visual pigments. Vitamin A is also recovered from the LAP pathway increasing the efficiency of the recovery process. Thus, the important processes of phagocytosis and the visual cycle converge as the recovery of all-trans ROL for 11-cis RAL synthesis in the RPE is aided by the LAP pathway. Blood absorption is also an important source of Vitamin A for vision. *RPE, retinal pigment epithelium; PR, photoreceptors; POS, photoreceptor outer segments; 11-cis RAL, 11-cis retinal; All-trans ROL, All-transretinol; L, lysosome*.

An important function of the RPE is the visual cycle, a biochemical process that supports vision by recycling the chromophore 11-*cis* retinal (11-*cis* RAL). The LAP pathway plays an important role in this process and represents an important enhancement to the chromophore recycling process. The visual cycle begins following the absorption of light by visual pigments (opsins) containing 11-*cis* RAL, converting it to the all-*trans* configuration. All-*trans* RAL is released from opsin and reduced to all-*trans* ROL (retinol, vitamin A) by retinoid dehydrogenases. All-*trans* ROL is transported to the RPE where it is esterified by lecithin retinol acyltransferase to form all-*trans* RE (retinal ester). The isomerohydrolase RPE65 converts all-*trans* RE to 11-*cis* ROL, which is then oxidized to 11-*cis* RAL and transported back to photoreceptors to complete the visual cycle (Figure 7). The primary sources of vitamin A for the RPE visual cycle were thought to be the diffusion from photoreceptors after light absorption and exogenous retinol absorbed from the blood [325]. However, we now know that the LAP pathway is a third source of retinol critical for vision [228]. This was discovered when it was observed that in the absence of ATG5 (and LAP) visual responses in rods and cones (as measured by electroretinogram) were diminished, with no overt loss of PRs. This phenotype was not present in ULK1-deficient animals, where LAP proceeds normally. It was also observed that key visual cycle intermediates such as 11-*cis*, all-*trans*, and 13-*cis* RAL were significantly reduced when LAP was defective. Supplementation of LAP-deficient mice with the 11-*cis* RAL analogue 9-*cis*-RAL restored vision demonstrating that LAP was responsible for recovering a portion of the vitamin A for visual cycle. It was this decrease in retinoids from defective LAP that led to the reduced visual responses. Therefore, the recovery of this vitamin A from recycled photoreceptor tips by LAP increases the efficiency of the process. It was also noted that vision loss in the absence of LAP was cumulative as the reduction in retinoids and electroretinogram responses was not observed in younger animals [228,326], but became manifested several months into adulthood [228]. Decreases in autophagy-related process has been suggested as a sign of aging [5]. Perhaps reduced vitamin A recovery due to defective LAP is a reason why elderly individuals have difficulties with night vision and supplementation with nutritional vitamin A can improve this condition [327]. Unfortunately, the mechanisms for vitamin A recovery by LAP have not been fully elucidated. In addition, it is not known if other phagocytic cells in the eye, such as microglial, Müller glial, or astrocytes perform LAP. This may be important particularly for Muller glial cells as they perform the cone visual cycle [328]. However, the description of LAP as a separate pathway from canonical autophagy gives us a starting point for therapeutic intervention for diseases linked to disruptions in these processes.

## 13. Conclusions

In summary, autophagy plays an essential role in the physiology, pathophysiology and response to disease in all ocular tissues. Mutations in autophagy-related genes have been linked to eye diseases, in particular cataracts, glaucoma, and corneal dystrophy (summarized in Table 1). Reciprocally, as summarized in Table 2, alterations in autophagy and lysosomal pathway are a common finding in all diseases of the eye. Moreover, LAP is critical in promoting the visual cycle. A significant progress has been made in understanding some of the mechanisms by which autophagy exerts its effects, especially with the generation of tissue-specific transgenic mice (Table 3). There are still unresolved issues, which have been identified in each specific section. Taken together, much work remains to understand the specific upstream activators of autophagy and the downstream effects of its activation in each of the ocular tissues; the interaction between autophagy and other degradative systems (PLAAT, proteasome, unfolded response), as well as the effect of aging. A better understanding of the interrelationship between autophagy dysfunction, cellular senescence, oxidative stress, and inflammatory phenotype (i.e in AMD and glaucoma) with aging would help to develop protective strategies targeting autophagy to alleviate. In this regard, significant care must be demonstrated when studying autophagy in the ocular tissues, particularly as to the reference points and controls, and in determining whether the effect of autophagy modulation is positive or negative. There is no “one-size-fits-all” approach, with changes in autophagy levels having the potential to be either beneficial or detrimental, depending on the exact context of the disease state. Future work must be aimed at elucidating the specific ways in which autophagy modulation can be used to improve visual function with aging and in disease.

**Table 1.** Alterations in autophagy-related genes linked to eye diseases

Symbol	Function	Phenotypic effect	Disease
OPTN	autophagy receptor, mitophagy, autophagosome formation	RGC loss, thinning of the retina, mitochondrial fission	glaucoma [195-197]
TBK1	phosphorylation of OPTN, mitophagy	unknown	glaucoma [195-197]
CRYAA, CRYAB	reduced autophagy	accumulation autophagic substrates	cataracts [105-108]
FYCO1	Rab7 effector; regulates transport of microtubules, intracellular quality control	accumulation of p62, crystallins	congenital cataracts [114]
CHMP4B	component of ESCRT system, autophagosome formation	severe cellular damage	congenital cataracts [121]
EPG5	Rab7 effecto- Autophagosome maturation		congenital cataracts [122]
TBC1D20	GTPase-activating protein (GAP) for Rab1	disrupted autophagy flux and delayed differentiation in lens fibers	cataracts [128]
TDRD7	TBC1D20 regulator	disrupted autophagy flux and delayed differentiation in lens fibers	cataracts [129]
RRAGA	mTORC1 regulator	reduced autophagy	autosomal dominant cataracts [133]
PIKFYVE	produces PtdIns(3,5)P_2_	extensive vacuolization	autosomal dominant cataracts [135] corneal dystrophy [136]
GJA8	vesicle-mediated transport	impaired autophagy and terminal lens fiber differentiation	autosomal recessive cataracts [138]
LACRT	FOXO-Atg/promotes autophagy flux		dry eye [36-38]

**Table 2.** Summary of the Proposed Roles of Autophagy in Eye Tissues and Autophagy Status in Disease

Tissue	Role of Autophagy	Disease	Autophagy in Disease
TM/SC	Mechanical forces /IOP homeostasis TGF-signaling/fibrosis Cellular senescence Anti-oxidant	Ocular Hypertension	Impaired autophagy flux Constitutive mTOR activation (human TM primary cultures) Autophagy activation in OHT animal models
Lens	Organelle degradation? Maintenance of lens homeostasis and transparency Intracellular quality control of the lens	Cataracts	Dysregulated (insufficient) autophagy Accumulation of SQSTM1 positive aggregates
RGC/ON	Conflicting: neuroprotection and neuronal death	Glaucoma	Decrease autophagy flux Autophagy activation in some studies, decrease autophagy in others Defective mitophagy in ON
Cornea	Corneal epithelial regeneration/limbal Cell homeostasis Transparency corneal layers Defense against pathogen infection	Dry eye	Autophagy dysfunction, decreased LACRT
		Keratococus	Impaired autophagic flux (decreased LC3II & LAMP1, increase p62)
		Granular corneal dystrophy	Impaired autophagic flux (decreased TFEB?)
		Macular corneal dystrophy	Reduced autophagy activation
		Fuchs corneal dystrophy	Overactivation of mitophagy (increased LC3 and DRAM1)
		Congenital endothelial dystrophy	Autophagy dysfunction (decreased nuclear translocation of TFEB)
RPE	Cellular quality control RPE homeostasis Maintenance of visual pigment balance OS phagocytosis Metabolic homeostasis	AMD	Reduced autophagy activity Accumulation of lipofuscin
PRs	Vision cycle Maintain cellular homeostasis Response to environmental stressors	RD, RP	Autophagy activation

**Table 3.** Major Eye Phenotypes of Mouse Models with Modulated Autophagy Activity

	Cornea	Lens	TM/SC	RGC	RPE	PR
*Ambra1+/gt*				decrease RGC survival following acute OHT [185, 193, 194]	lipofuscin accumulation, increased ROS, higher sensitivity to sodium iodate model [224]	increased protein aggregation, altered mitochondrial function [224]
*Atg4b^−/−^*				decrease RGC survival following ON crush [191]		
*Atg5-/-*		accumulation of autophagic substrates and SQSTM1, age- related cataracts [11]		decrease RGC survival following ON crush [191]	uneven RPE thickness, hyper- /hypotrophy, choroidal neovascularization, disrupted visual cycle, inflammation, senescence [264]	improved PR structure and function [278,279]
*Atg7-/-*					uneven RPE thickness, hyper- /hypotrophy, choroidal neovascularization, disrupted visual cycle, inflammation, senescence [264]	
*Becn1^+/-^*	decreased proliferation of corneal epithelial limbal stem and transit- amplifying cells [31]					
*Chmp4b^−/−^*		severe cellular damage, congenital cataracts [121]				
*Fyco1^−/−^*		accumulation of SQSTM1, age-related cataracts [114]				
GFP-LC3			higher IOP [154,168]	RGC loss, axonal degeneration [154, 168]		
*Lc3b-/-*					Chronic inflammation, lipid deposition and increased cholesterol adducts, decreased oxidative metabolism, decreased neuroprotection, metabolic dysregulation [230]	Decreased PR function [231]
OPTNE50K				mitochondrial fission, mitophagy, RGC loss, glaucoma [203]		
*Pik3c3^−/−^*		accumulation of autophagic substrates and SQSTM1, enlargement of endolysosomes, congenital cataracts [11]				
*Tbc1d20^−/−^*		disrupted autophagic flux, delayed differentiation of lens fibers, cataracts [128]				
*Tdrd7^−/−^*		disrupted autophagic flux, delayed differentiation of lens fibers, cataracts [129]				

## Supplementary Material

Supplemental Material
